# Nucleophilic Attack of Azide at Electrophilic Azides: Formation of N_6_ Units in Hexazene and Aminopentazole Derivatives†

**DOI:** 10.1002/anie.202003010

**Published:** 2020-04-30

**Authors:** Klaus Banert, Tom Pester

**Affiliations:** ^1^ Chemnitz University of Technology Organic Chemistry Strasse der Nationen 62 09111 Chemnitz Germany

**Keywords:** azides, isotopic labeling, nitrogen heterocycles, reaction mechanisms, reactive intermediates

## Abstract

With the help of selective ^15^N labeling experiments, it has been confirmed that nucleophilic attack of azide at iminium‐activated organic azides leads to short‐lived hexazene intermediates. Such species do not only tend to a cleavage reaction with formation of N‐azido compounds, but also undergo ring closure to generate unprecedented amidino‐functionalized pentazoles. Thus, treatment of the parent Vilsmeier reagent with two equivalents of sodium azide creates an aminopentazole derivative as the main product, which is easily characterized by NMR spectroscopy.

## Graphical abstract

**Simple substrates**, all commercially available, lead to previously unknown aminopentazole derivatives. By using Na^15^N_3_, the heterocyclic product with six‐fold ^15^N labelling is easily accessible, which enabled structure identification by ^15^N NMR spectroscopy without comparison with known data.

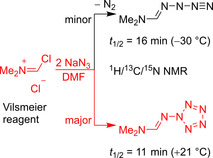

Initial attempts to generate an all‐nitrogen five‐membered ring were performed more than a hundred years ago.^1^ But the first report with evidence of the arylpentazole **2** dates back from the year 1956 when Huisgen and Ugi described experiments with such intermediate species in the transformation of aryldiazonium salts **1** to prepare aryl azides **3** (Scheme 1 a).^2^ Later, isolation and even characterization of pentazole **2** (Ar=4‐Me_2_NC_6_H_4_) with the help of single‐crystal X‐ray diffraction analysis were successful.^3^ However, attempted modification of the aryl group of **2** have usually resulted in destruction of the pentazole ring, which degraded rapidly at ambient temperature with evolution of dinitrogen.^4^ To our knowledge, the reaction of substrates **1** with azide salts proves to be the only method for the synthesis of pentazoles, and aryl derivatives of type **2** are the only representatives which could be prepared so far.^5^

**Scheme 1:**
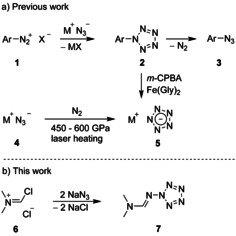
Synthesis of penzatoles **2** and **7** as well as pentazolate salts **5**.

After a long period of unsuccessful attempts and hundreds of experiments, reaction conditions were recently found for the cleavage of arylpentazole **2** (Ar=3,5‐dimethyl‐4‐hydroxyphenyl) in the presence of *m*‐chloroperbenzoic acid (*m*‐CPBA) and ferrous bisglycinate [Fe(Gly)_2_].^6^ This transformation led to the salt **5** (M=NH_4_) and effected a breakthrough in pentazolate chemistry and also in the synthesis of other salts of type **5**.^7^ Nearly simultaneously, the products **5** (M=Cs, Li) were generated by laser heating of alkali azides **4** in the presence of dinitrogen under very high pressure (Scheme 1 a).^8^ Currently, polynitrogen compounds, such as pentazoles and pentazolate salts, attract attention because they are assumed to have important applications as high energy density materials (HEDMs).^9^

Herein, we report an unprecedented synthesis of the aminopentazole derivative **7**, which is available by treating the commercial Vilsmeier reagent **6** with sodium azide (Scheme 1 b). The product **7** is probably formed via cyclization of a short‐lived hexazene derivative that is generated by nucleophilic attack of azide at an iminium‐activated organic azide. This assumption is based on mechanistic studies with other chloroiminium salts (for example, see **8**) exposed to sodium azide.

Our approach to prepare aminopentazole derivatives was based on the work of Balli and co‐workers,^10^ who synthesized the 2‐azidobenzothiazolium salt **9** by treating the precursor **8** with one equivalent of sodium azide (Scheme 2). The reaction of **9** with lithium azide in dimethylformamide led to the highly unstable *N*‐azido compound **13**. The salt **10**, which should establish an equilibrium with the covalent diazide **11**,^11^ and the hexazene^12^ derivative **12** were postulated as short‐lived intermediates to explain the formation of the unusual final product **13**.^10^

**Scheme 2:**
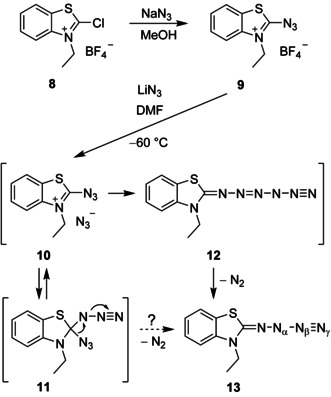
Generation of the *N*‐azido compound **13**.

Quite recently, it was shown that the structures of several *N*‐azidoamines, which were previously reported in the literature, are not correct and have been revised.^13^ Thus, we assumed that structural verification of the *N*‐azido compound **13** might be useful since the former characterization was mainly based on IR data^10^ owing to the low stability of this substance. The reaction mechanism including nucleophilic attack of azide at the terminal nitrogen atom of the iminium‐activated azido group of **10** and formation of intermediate **12** is plausible but also an unprecedented case. We thought that migration of an azido group of diazide **11** accompanied by liberation of dinitrogen may alternatively lead directly to **13** without the creation of **12**. If hexazene intermediate **12** is really generated, however, cyclization to produce the corresponding aminopentazole derivative is possibly observable at low temperature. This assumption is based on the well‐known ring closure of pentazenes to yield pentazoles.^5^, ^14^

When we treated the substrate **9** in d_7_‐DMF with hexadecyltributylphosphonium azide (Scheme 3), which includes a ^15^N label at one of the terminal nitrogen atoms (98 %),^15^ we obtained the desired *N*‐azido compound ^15^N_3_‐**13** quantitatively (^1^H NMR spectroscopy at −60 °C). The ^15^N NMR spectrum of this product indicated that the label was distributed among the imino group as well as N‐β and N‐γ of the azido group. Both signals of the azido group were accompanied by small doublets, which revealed a direct coupling of the two nitrogen atoms with ^1^ *J*(^15^N,^15^N)=9.3 Hz. These results show that the equilibration of isotopically labeled intermediates **10** and **11** led to such species with two, one, or zero ^15^N atoms. The distribution of the ^15^N label, observed in ^15^N_3_‐**13**, is only compatible with formation of **13** via cleavage of **12**.^16^ In the alternative case with liberation of dinitrogen from **11**, the isotopically isomeric product ^15^N_3_‐**13′** should be generated. We treated **13** and ^15^N_3_‐**13** with cyclooctyne^17^ in order to confirm the *N*‐azido structure and the distribution of the ^15^N label. The product ^15^N_3_‐**14** was formed quantitatively (^1^H NMR spectroscopy) and led to ^15^N NMR signals at *δ*=−158.7, −48.7, and −44.7 ppm, which were compared with the ^15^N NMR spectrum of **14** measured with natural abundance (Scheme 3). The signals of ^15^N_3_‐**14** with *δ*=−48.7 and −44.7 ppm were accompanied by small doublets with ^1^ *J*(^15^N,^15^N)=18 Hz. Whereas the 2D‐^15^N,^1^H shift correlation spectrum of **14** showed cross signals for N‐3′/4′‐H as well as N‐1′/9′‐H, the corresponding spectrum of ^15^N_3_‐**14** indicated the former cross signal only, and a correlation of the 9′‐H signal with nitrogen signals was not observed.

**Scheme 3:**
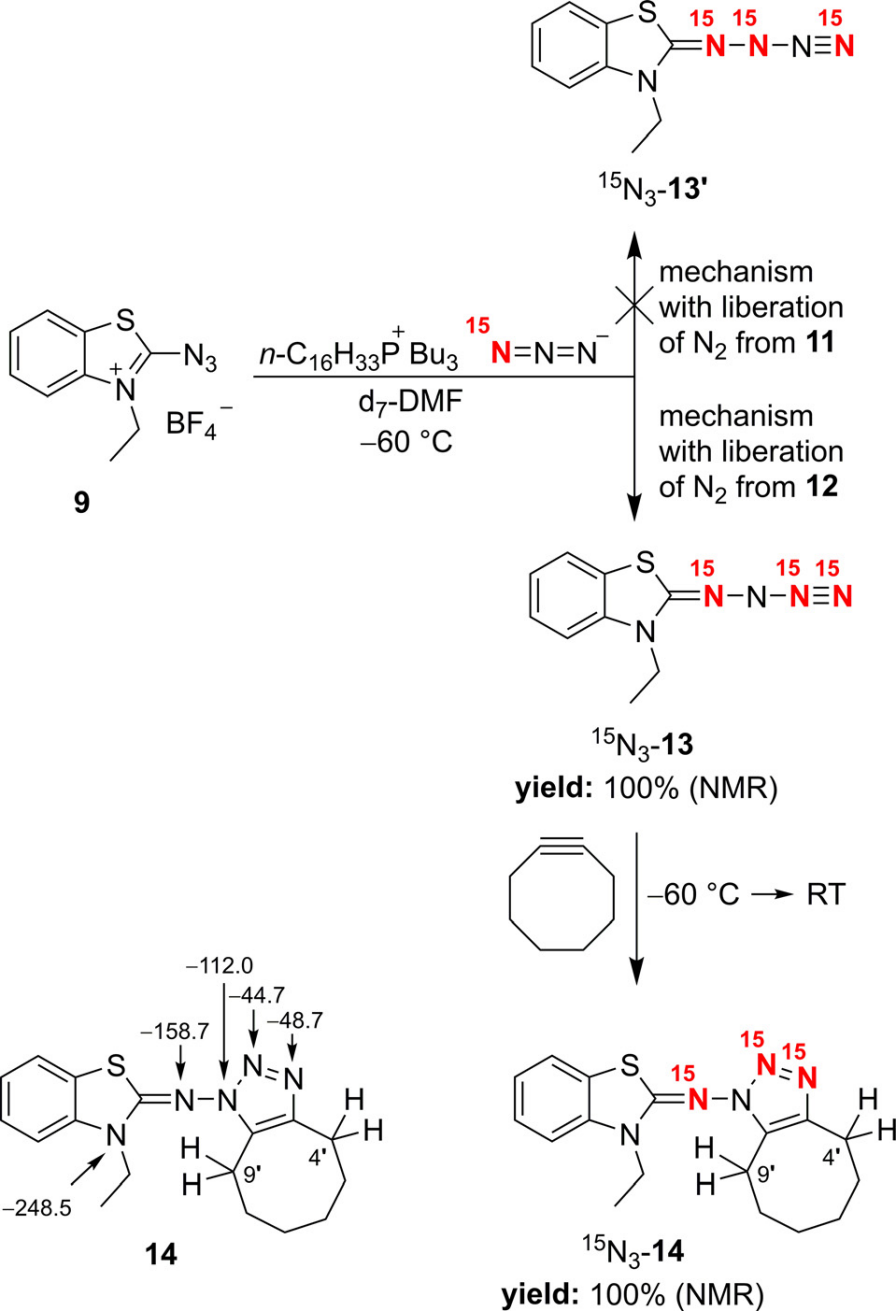
Synthesis of the ^15^N‐labeled compounds **13** and **14** as well as ^15^N NMR chemical shifts (*δ*) of **14**.

Our investigations allow the drawing of the following interim conclusions: The ^15^N NMR data and the trapping reaction of **13** with the help of cyclooctyne confirm the *N*‐azido structure of **13**. Moreover, it is demonstrated that nucleophilic attack of azide at iminium‐activated organic azides is possible since **13** was generated from **9** via hexazene derivative **12**. Disappointingly, cyclization of short‐lived **12** to create an aminopentazole derivative was not observed.

When we planned and performed final experiments to additionally verify the ^15^N NMR data of **13** by synthesizing ^15^N_4_‐**13** from precursor **8** and two equivalents of fully labeled sodium azide (Na^15^N_3_, 98 %), we obtained not only the desired product ^15^N_4_‐**13**, but also the surprising compounds **15**, ^15^N_6_‐**7**‐d_7_, and ^15^N_4_‐**17**‐d_7_ (Scheme 4). Clearly, the formation of the unexpected products was connected with a halogen/oxygen exchange reaction of the solvent d_7_‐dimethylformamide and the substrate **8** to form the deuterated Vilsmeier reagent **6**‐d_7_.^18^ We assumed that **6**‐d_7_ was transformed into the hexazene derivative ^15^N_6_‐**16**‐d_7_ by double nucleophilic attack of azide, which is similar to the creation of **12** from **8**. Whereas **12** exclusively underwent liberation of dinitrogen to produce **13**, the short‐lived intermediate ^15^N_6_‐**16**‐d_7_ preferred cyclization leading to the aminopentazole derivative ^15^N_6_‐**7**‐d_7_, and the *N*‐azido compound ^15^N_4_‐**17**‐d_7_ was formed as a minor product. When we analogously treated Vilsmeier reagent **6** with fully ^15^N labeled sodium azide or with selectively labeled Na^15^N=N=N, we obtained ^15^N_6_‐**7** and ^15^N_4_‐**17** or ^15^N_4_‐**7** and ^15^N_3_‐**17**, respectively. If d_7_‐dimethylformamide was used as solvent for the reaction of **6** with fully ^15^N labeled sodium azide, the halogen/oxygen exchange reaction of the deuterated solvent and **6** caused generation of **6**‐d_7_ and thus creation of ^15^N_6_‐**7**‐d_7_ and ^15^N_4_‐**17**‐d_7_. In all cases, the molar ratio of **7** to **17** was approximately 2:1.

**Scheme 4:**
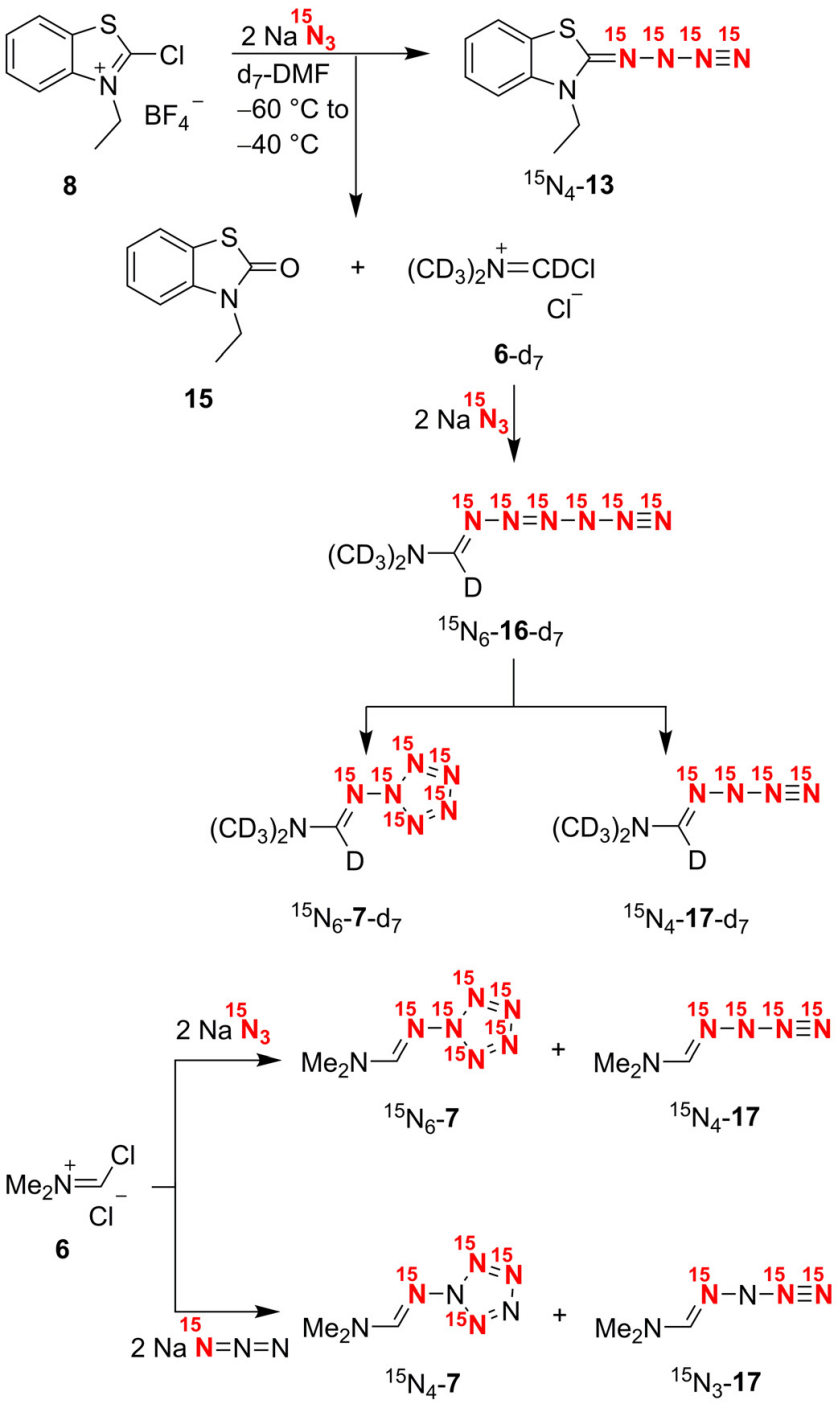
Synthesis of aminopentazole derivatives **7** from Vilsmeier reagent **6** and sodium azide.

The *N*‐azido compound **17** can easily be handled in solution at −40 °C; however, rapid decay with a half‐life *t*_1/2_ of approximately 16 min, which was measured by collecting the liberated dinitrogen gas, was observed at −30 °C.^16^ Hence, **17** is significantly less stable than **13**, and consequently, **17** did not undergo a clean trapping reaction with cyclooctyne.^19^ On the other hand, solutions of the aminopentazole derivative **7** can be utilized for NMR spectroscopy at +10 °C, and a half‐life *t*_1/2_ of around 11 min was roughly estimated at 21 °C. The identification of **7** and **17** was mainly based on NMR spectroscopy and especially ^15^N NMR data. The ^15^N NMR data of ^15^N_4_‐**17** and those of ^15^N_4_‐**13** are very similar.^16^ The ^15^N NMR chemical shifts and the ^15^N,^15^N coupling constants of ^15^N_6_‐**7** and ^15^N_4_‐**7** are in excellent agreement with those published for several other pentazoles.4b, 5a, 20 To our knowledge, ^15^N_6_‐**7** is the first pentazole derivative in which all members of the ring are labeled by ^15^N atoms. Therefore, there is no need to compare with known data of other pentazoles since the coupling patterns alone are unequivocal proof of the structure with an all‐nitrogen five‐membered ring connected with a sixth nitrogen atom. As depicted in Figure 1, the imine nitrogen atom couples with N‐1 (^1^ *J*=−14.3 Hz), and a direct coupling with N‐2 and N‐5 is responsible for the additional triplet splitting of the N‐1 signal (^1^ *J*=−18.0 Hz). Other triplet splittings were detected for the N‐1 signal by geminal coupling with N‐3 and N‐4 (^2^ *J*=0.8 Hz) and also for the imine signal by geminal coupling with N‐2 and N‐5 (^2^ *J*=2.2 Hz). Finally, N‐2, N‐5, N‐3, and N‐4 create an AA′XX′ system, which was analyzed by iterative simulation of the ^15^N NMR spectrum.^16^

**Figure 1:**
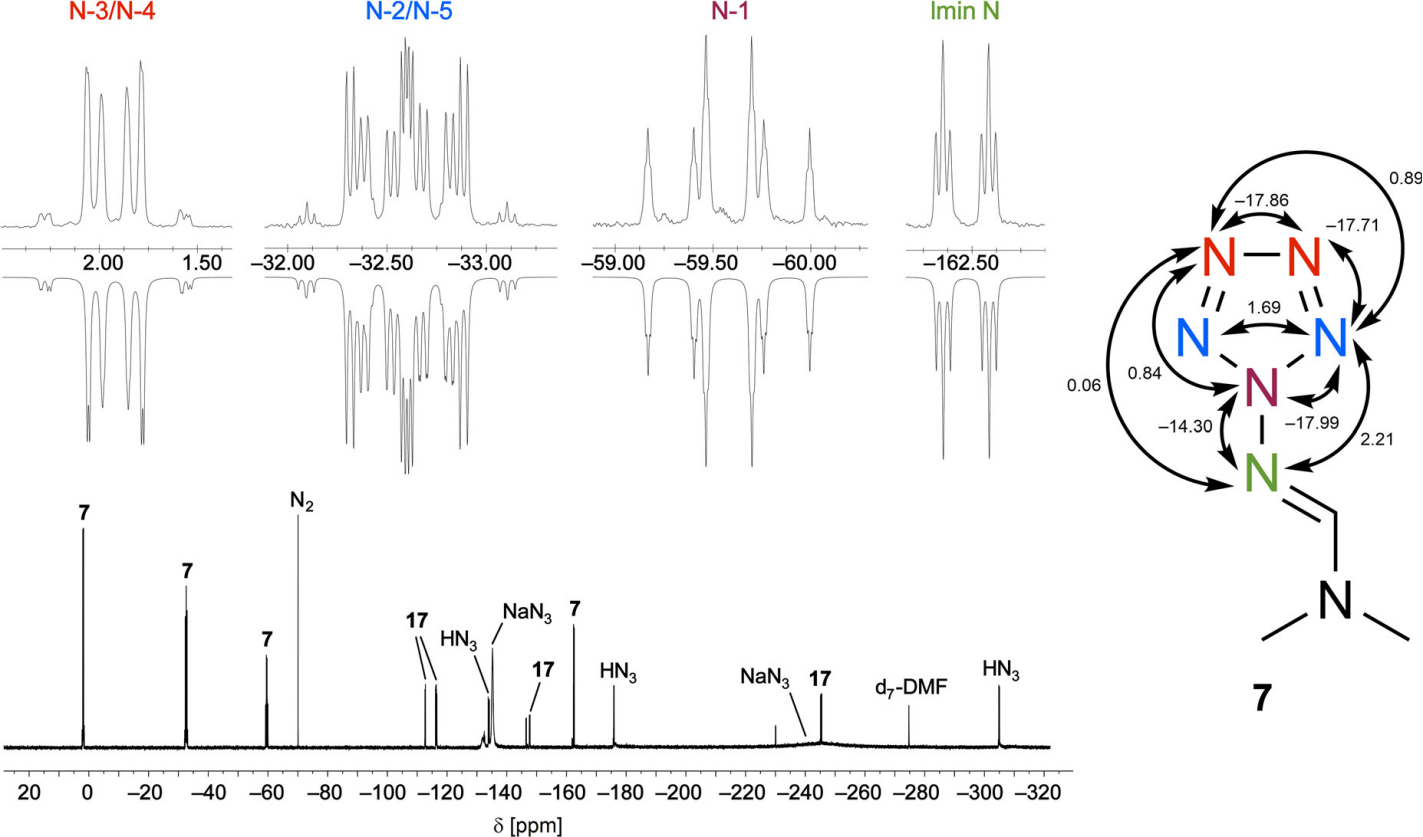
^15^N NMR spectrum of ^15^N_6_‐**7** and ^15^N_4_‐**17** in d_7_‐DMF measured at −60 °C (61 MHz, reference MeNO_2_ with *δ*=0; *J* values in Hz*)*.

In conclusion, we confirmed the nucleophilic attack of azide at the terminal nitrogen atom of iminium‐activated organic azides. Clearly, the resulting short‐lived hexazene derivatives undergo not only a cleavage reaction to generate *N*‐azido compounds, but also a cyclization leading to unprecedented aminopentazole^21^ structures. This simple access to amidino‐substituted pentazoles is remarkable, especially as the precursor **6** is a commercial substance known as the Vilsmeier reagent and isolated in 1959 for the first time.^22^ Currently, we investigate whether the new approach to functionalized pentazoles can be transferred to other chloroiminium substrates. Preliminary experiments have shown that pentazolate salts are also available by similar reactions. Moreover, we assume that the decay of **7** and **17** will offer an access to dimethylaminomethylidene, a rarely studied carbene.^23^ This expectation is based on the known decomposition reaction of **13**, which led to the corresponding short‐lived benzothiazol‐2‐ylidene.10g

## Conflict of interest

The authors declare no conflict of interest.

## Supporting information

As a service to our authors and readers, this journal provides supporting information supplied by the authors. Such materials are peer reviewed and may be re‐organized for online delivery, but are not copy‐edited or typeset. Technical support issues arising from supporting information (other than missing files) should be addressed to the authors.

Supplementary
