# Supplementary material for: Nucleophilic Attack of Azide at Electrophilic Azides: Formation of N6 Units in Hexazene and Aminopentazole Derivatives
Source: Angew Chem Int Ed Engl. 2020 Apr 30;59(30):12315–20. doi: 10.1002/anie.202003010 (PMC7383498; doi:10.1002/anie.202003010)
Supplement: Supplementary file 1 — Supplementary [file ANIE-59-12315-s001.pdf]

Angewandte Chemie
Eine Zeitschrift der Gesellschaft Deutscher Chemiker

Supporting Information

Nucleophilic Attack of Azide at Electrophilic Azides: Formation of N_6 Units in Hexazene and Aminopentazole Derivatives**

Klaus Banert and Tom Pester*

anie_202003010_sm_miscellaneous_information.pdf

Table of Contents

Table of Contents 2

1. General 3

2. Apparatus 3

3. Chemicals 3

3.1. Purchaseble chemicals 3

3.2. Non-purchaseble chemicals 3

4. Reactions of the benzothiazole derivatives **8** and **9** 4

4.1. Synthesis of ^{15}N-labeled **13** in d_7-DMF 4

4.2. Distribution of ^{15}N-labels in **13** via 4.1. 5

4.3. Trapping reaction of **13** with cyclooctyne 6

5. Reaction of Vilsmeier reagent **6** and determination of the half-life of compund **7** and **17** 7

6. Spectra 8

7. References 26

8. Author Contributions 26

1. General

CAUTION! All experiments dealing with the synthesis of small azides should be performed with extra safety arrangements because of their potential explosive character. Extra safety arrangements include a safety windowpane and shatter protection gloves. Isolation of highly explosive compounds should be avoided; always handle such substances in diluted solutions. Furthermore, the interaction of chlorinated solvents like DCM with azide salts carries the risk of the formation of highly explosive organic azides such as diazidomethane.

All reactions dealing with air- or moisture-sensitive compounds were carried out in a dry reaction vessel under a positive pressure of argon. Air- and moisture-sensitive liquids and solutions were transferred via a syringe. All reactions were carried out with freshly distilled, in some cases, dry solvents. Anhydrous solvents were distilled immediately before use.

2. Apparatus

NMR spectra were recorded with an Avance *NEO* 600 FT spectrometer (Bruker Corp., Billerica, MA) operating at 600 MHz for ^{1}H NMR, 150.9 MHz for ^{13}C NMR and 60.8 MHz for ^{15}N NMR. ^{1}H NMR and ^{13}C NMR signals were referenced with the help of the solvent signals and recalculated relative to TMS. Data are presented as follows: chemical shift, multiplicity (s = singlet, d = doublet, t = triplet, q = quartet, quint = quintet, sext = sextet, sept = septet, m = multiplet, br = broad), coupling constants in hertz (Hz), followed by the number of hydrogen atoms. Assignments of NMR signals were further supported by NOE, COSY, HSQC, and HMBC 2D-NMR methods and also by comparison of the data of homologous compounds in several cases. Signal assignment was omitted if it was unclear. Mass spectra were obtained from a micrOTOF spectrometer (Bruker Corp., Billerica, MA) utilizing an electrospray-ionization technique. Quantitative elementary analyses were performed on a vario Micro cube (Elementar Analyensysteme GmbH, Langenselbold, HE, Germany). Melting points (mp) were measured by the BOETIUS method on a heating apparatus from VEB Analytik Dresden PHMK 74/0032.

3. Chemicals

3.1. Purchaseble chemicals

Triethyloxonium tetrafluoroborate, and oxalyl chloride were obtained from Alfa Aesar (Germany). Dry DMF, and Vilsmeier reagent (also prepared from dry DMF and oxalyl chloride, see paragraph below) were obtained from Sigma-Aldrich (Germany). d_7-DMF was obtained from Deutero GmbH (Germany). Unlabeled sodium azide was obtained from Novasep (Germany). $^{15}N_1$-sodium azide (98%), and $^{15}N_3$-sodium azide (98%) were purchased from Cambridge Isotope Laboratories, Inc. (MA, USA). 2-Chlorobenzothiazole was purchased from Fluorochem (UK). All purchasable chemicals were used without further purification.

3.2. Non-purchaseble chemicals

8[10a], **9**[10a], cyclooctyne[17], and QN_3[S-1] (Q = n-$C_{16}H_{33}$(n-Bu)$_3P^+$) were prepared according to the reported literature.

Preparation of Vilsmeier reagent **6**:

In a Schlenk tube, dry DMF (degassed, 0.3 mL, 142 mg, 2 mmol) was dissolved in dry *n*-pentane (10 mL) and treated slowly (Caution! Strong gas evolution!) under argon atmosphere at room temperature with oxalyl chloride (1 mL, 1.48 g, 12 mmol, 6 eq.). The solution was stirred until the gas evolution deceased. Over this period (approx. 15 min), additional dry *n*-pentane (10 mL) was added. The solution was removed via syringe and the remaining solid was washed with dry diethylether (5 x 10 mL) and subsequently dried under reduced pressure. **6** was obtained as a white solid (250 mg, 2 mmol, quant.).

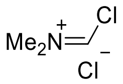

6

4. Reactions of the benzothiazole derivatives 8 and 9

4.1. Synthesis of ^{15}N-labeled 13 in d_7-DMF

Method a)

In a NMR tube equipped with small magnetic stirring bars (5 piec., 2 mm x 5 mm), a solution of **9** (34.2 mg, 0.1 mmol) in d_7-DMF (0.35 mL) was cooled to –60 °C and treated with a precooled solution of $^{15}N_1$-QN_3 (50 mg, 0.1 mmol, 1 eq.) in d_7-DMF (0.35 mL). The mixture was stirred for 1 h at –60 °C to –40 °C. After removing the stirring bars, the solution was directly measured. Compound $^{15}N_3$-**13** was obtained in quantitative yield (^{1}H NMR). (spectra: See section 6, page S-8)

Method b)

In a NMR tube equipped with small magnetic stirring bars (5 piec., 2 mm x 5 mm), a suspension of $^{15}N_3$-NaN_3 (24 mg, 0.37 mmol, 2.1 eq.) in d_7-DMF (0.35 mL) was cooled to –60 °C and treated with a precooled solution of **8** (50 mg, 0.18 mmol) in d_7-DMF (0.35 mL). The mixture was stirred for 1 h at –60 °C to –40 °C. After removing the stirring bars, the solution was directly measured. Compound $^{15}N_4$-**13** was obtained as a mixture with **15**, $^{15}N_6$-**7**, and $^{15}N_4$-**17** (yield: 57%, ^{1}H NMR).

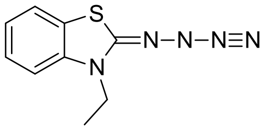

13

^{1}H NMR (600 MHz, **d_7-DMF**, –**60 °C):** δ [ppm] = 1.26 (t, 3H, $^3J_{HH}$ = 6.8 Hz, –N–CH_2–C$\underline{H}_3$), 4.11 (q, 2H, $^3J_{HH}$ = 6.8 Hz, –N–C$\underline{H}_2$–CH_3), 7.10–7.18 (m, 1H, H-5), 7.39–7.43 (m, 2H, H-6/H-7), 7.77–7.82 (m, 1H, H-4).

^{13}C NMR (150.9 MHz, **d_7-DMF**, –**60 °C):** δ [ppm] = 12.0 (q, –N–CH_2–$\underline{C}H_3$), 39.2 (t, –N–$\underline{C}H_2$–CH_3), 110.5 (d, C-6), 122.1 (d, C-5), 123.41 (d, C-4), 123.43 (s, C-7a), 127.6 (d, C-7), 140.4 (s, C-3a), 156.3 (s, C-2).

^{15}N NMR (60.8 MHz, **d_7-DMF**, –**60 °C**, $^{15}N_4$-**13):** δ [ppm] = –242.09 (dd, $^1J(N_\alpha N_\beta)$ = 15.1 Hz, $^1J(N_\alpha N_{imine})$ = 9.7 Hz, N_α), –155.45 (ddd, $^1J(N_{imine}N_\alpha)$ = 9.7 Hz, $^2J(N_{imine}N_\beta)$ = 4.0 Hz, $^3J(N_{imine}N_\gamma)$ = 1.4 Hz, N_{imine}), –119.68 (ddd, $^1J(N_\beta N_\alpha)$ = 15.1 Hz, $^1J(N_\beta N_\gamma)$ = 9.3 Hz, $^2J(N_\beta N_{imine})$ = 4.0 Hz, N_β), –106.88 (dd, $^1J(N_\gamma N_\beta)$ = 9.3 Hz, $^1J(N_\gamma N_{imine})$ = 1.4 Hz, N_γ).

4.2. Distribution of ^{15}N-labels in 13 via 4.1.

The following mechanism is explaining the distribution of the ^{15}N-labels in compound **13** obtained via procedures 4.1. Nitrogens in red and in blue are ^{15}N-labeled. It is important that labels with the **same** color cannot exist at same time in the same molecule. Only ^{15}N-labels in different colors can be found in one molecule at the time. This is because of the nature of the used, single labeled Q^{15}**N**=N=N. After getting covalently bond, the label can be found in N_α (50%) or N_γ (50%).

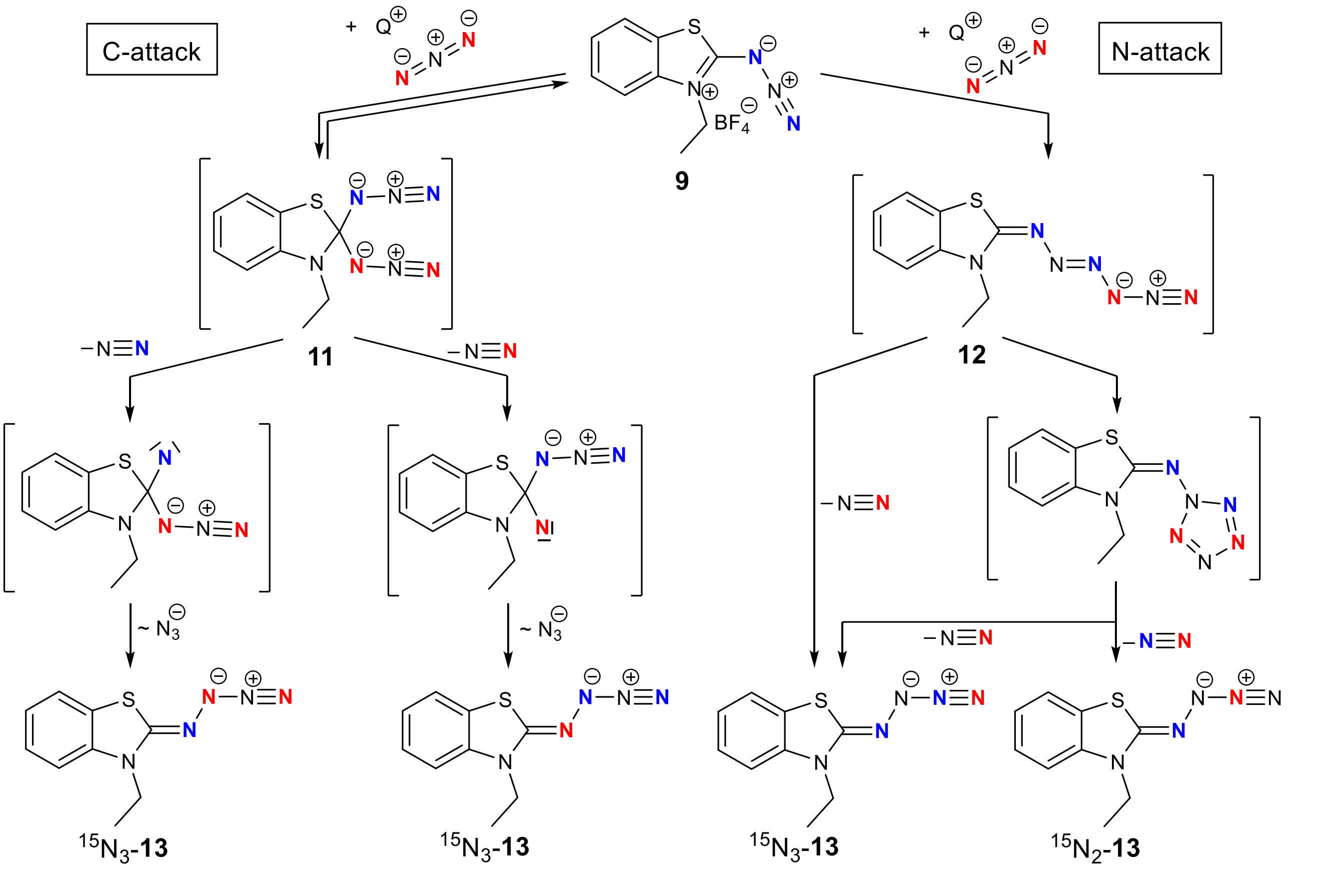

Via a C-attack of azide, labels can be found at N_{imine}, N_α and N_γ. A N-attack leads to labels in N_{imine}, N_β and N_γ. The latter case was found. (spectrum: See section 6 on page S-10)

4.3. Trapping reaction of 13 with cyclooctyne

Method a)

The solution obtained via 4.1. was treated with cyclooctyne (32.5 mg, 0.3 mmol, 3 eq.) in d_7-DMF (0.2 ml) at –40 °C and then warmed up overnight to room temperature. Compound $^{15}N_3$-**14** was obtained in quantitative yield (^{1}H NMR). (spectra: See section 6, page S-13)

Method b)

In a Schlenk tube under argon atmosphere, **9** (114.2 mg, 0.4 mmol) was dissolved in dry acetonitrile (4 mL) and treated with LiN_3 (76.4 mg, 1.6 mmol, 4 eq.) at –60 °C to –40 °C. After 3 h at this temperature, the evolution of dinitrogen deceased and cyclooctyne (84.5 mg, 0.8 mmol, 2 eq.) was added to the solution. The reaction mixture was hold for 30 min at –30 °C and then the cooling bath was removed. After 1 h at room temperature, the suspension was filtered and the solid was extracted with DCM (3 x 5 mL). The volatiles of the combined organic phases were removed at reduced pressure ($6.8 \cdot 10^{-3}$ mbar). Compound **14** was obtained as a white to beige, partly crystalline, solid after flash column chromatography (silca gel 60, (EtOAc : DCM) = (1:1), R_F = 0.54, mp: 111–114°C, 88.3 mg, 0.27 mmol, yield: 69%).

Method c)

In a Schlenk tube under argon atmosphere, **8** (150 mg, 0.5 mmol) was dissolved at –60 °C in dry DMF (4 mL) and treated with $Na^{15}N_3$ (71.7 mg, 1.1 mmol, 2.1 eq.). After 1 h at this temperature the evolution of dinitrogen deceased and cyclooctyne (113.7 mg, 1.0 mmol, 2 eq.) was added to the solution. The reaction mixture was hold for 30 min at –30 °C and then the cooling bath was removed. After 1 h at room temperature, the volatiles were removed at reduced pressure ($6.8 \cdot 10^{-3}$ mbar) and the residue was extracted with DCM (4 x 5 mL). The combined organic phases were concentrated with a rotary evaporator. Flash column chromatography (silca gel 60, (EtOAc : DCM) = (1:1), R_F = 0.54) leads to pure $^{15}N_4$-**14**, which was obtained as a white, partly crystalline solid (mp: 112–115°C, 123.0 mg, 0.38 mmol, yield: 72%).

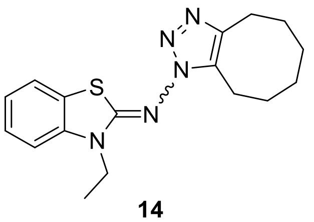

14

^{1}H-NMR (600 MHz, d_7-DMF, 25 °C): *δ* [ppm] = 1.40 (t, 3H, $^{3}J_{HH}$ = 7.1 Hz, –N–CH_2–C*H*$_3$), 1.45–1.50 (m, 2H, H-6‘), 1.50–1.55 (m, 2H, H-7‘), 1.73 (m, 2H, H-5‘), 1.82 (m, 2H, H-8‘), 2.87–2.90 (m, 2H, H-9‘), 2.89–2.91 (m, 2H, H-4‘), 4.37 (q, 2H, $^{3}J_{HH}$ = 7.1 Hz, –N–C*H*$_2$–CH_3), 7.22 (m, 1H, H-6), 7.46 (m, 1H, H-5), 7.49 (brd, $^{1}J_{HH}$ = 8.1 Hz 1H, H-4), 7.78 (brd, $^{1}J_{HH}$ = 7.6 Hz, 1H, H-7).

^{13}C-NMR (150.9 MHz, d_7-DMF, 25 °C): *δ* [ppm] = 11.7 (q, –N–CH_2–*C*H_3), 21.4 (brt, C-9‘), 24.9 (t, C-6‘), 25.1 (t, C-4‘), 25.9 (t, C-8‘), 26.5 (t, C-7‘), 28.7 (t, C-5‘), 39.8 (t, –N–*C*H_2–CH_3), 110.8 (d, C-4), 122.7 (d, C-7), 122.9 (d, C-6), 123.7 (s, C-7a), 127.5 (d, C-5), 130.1 (s, C-9a‘), 139.2 (s, C-3a), 143.3 (s, C-3a‘), 160.3 (s, C-2).

^{15}N-NMR (60.8 MHz, d_7-DMF, 25 °C, $^{15}N_4$-14): *δ* [ppm] = –245.0 (N-3, via ^{15}N,^{1}H shift correlation), –156.1 (d, $^{1}J(N_{imine}N\text{-}1')$ = 11.4 Hz, N_{imine}), –109.4 (ddm, ^{1}J(N-1‘N-2‘) = 18.4 Hz, $^{1}J(N\text{-}1'N_{imine})$ = 11.4 Hz, N-1‘), –41.1 (brtm, ^{1}J(N-2‘N-3‘) = 18.4 Hz N-3‘), –39.0 (t, $^{1}J(N\text{-}2'N_{imine})$ = 18.4 Hz, N-2‘).

^{1}H-NMR (600 MHz, $CDCl_3$, 25 °C): *δ* [ppm] = 1.41 (t, 3H, $^{3}J_{HH}$ = 7.2 Hz, –N–CH_2–C*H*$_3$), 1.46–1.53 (m, 2H, H-6‘), 1.53–1.58 (m, 2H, H-7‘), 1.76–1.82 (m, 2H, H-5‘), 1.82–1.89 (m, 2H, H-8‘), 2.81–2.89 (m, 2H, H-9‘), 2.94–3.02 (m, 2H, H-4‘), 4.23 (q, 2H, $^{3}J_{HH}$ = 7.2 Hz, –N–C*H*$_2$–CH_3), 7.09 (brd, $^{1}J_{HH}$ = 8.2 Hz, 1H, H-4), 7.12 (m, 1H, H-6), 7.34 (m, 1H, H-5), 7.46 (brd, $^{1}J_{HH}$ = 7.8 Hz, 1H, H-7).

^{13}C-NMR (150.9 MHz, $CDCl_3$, 25 °C): *δ* [ppm] = 11.8 (q, –N–CH_2–*C*H_3), 21.6 (t, C-9‘), 24.7 (t, C-4‘), 24.8 (t, C-6‘), 25.5 (t, C-8‘), 26.3 (t, C-7‘), 28.1 (t, C-5‘), 39.5 (t, –N–*C*H_2–CH_3), 109.5 (d, C-4), 122.14 (d, C-6), 122.21 (d, C-7), 124.0 (s, C-7a), 126.7 (d, C-5), 130.2 (s, C-9a‘), 138.6 (s, C-3a), 143.1 (s, C-3a‘), 160.5 (s, C-2).

^{15}N-NMR (60.8 MHz, $CDCl_3$, 25 °C, $^{15}N_4$-14): *δ* [ppm] = –248.4 (s, N-3, via ^{15}N,^{1}H shift correlation), –158.7 (d,$^{1}J(N_{imine}N\text{-}1')$ = 11.4 Hz, N_{imine}), –112.0 (ddm, ^{1}J(N-1‘N-2’) = 18.5 Hz, $^{1}J(N\text{-}1'N_{imine})$ = 11.4 Hz, N-1‘), –48.7 (br, N-3‘), –44.7 (t-br, *J* = 18 Hz, N-2‘).

HRMS: Calcd for $C_{17}H_{22}N_5S^+$ [M + H$^+$]: 328.1596, found 328.1593; Calcd for $C_{17}H_{21}{}^{15}N_4{}^{14}NSNa^+$ [M + Na$^+$]: 354.1291, found 354.1293.

EA: Anal. Calcd for $C_{17}H_{21}N_5S$: C, 62.36; H, 6.46; N, 21.39; S, 9.79. Found: C, 62.17; H, 6.39; N, 20.65; S, 9.83.

5. Reaction of Vilsmeier reagent 6 and determination of the half-life of compound 7 and 17

In a NMR tube equipped with small magnetic stirring bars (5 piec., 2 mm x 5 mm), pure **6** (18.8 mg, 0.15 mmol) was cooled to –60 °C and treated with a suspension of $^{15}N_3$-NaN_3 (20 mg, 0.31 mmol, 2.1 eq.) in d_7-DMF (0.7 mL). The mixture was stirred for 1 h at –60 °C to –40 °C. After removing the stirring bars, the solution was directly measured. Compound $^{15}N_6$-**7** was obtained as a mixture with $^{15}N_6$-**7**-d_7, $^{15}N_4$-**17**, $^{15}N_4$-**17**-d_7 and $^{15}N_3$-**HN_3**. (spectra: See section 6, page S-18)

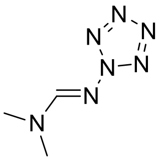

7

^{1}H NMR (600 MHz, **d_7-DMF**, –**60 °C):** *δ* [ppm] = 3.14 (s, 3H, (*Z*)–C$\underline{H}_3$), 3.35 (s, 3H, (*E*)–C$\underline{H}_3$), 9.04 (s, 1H, –C$\underline{H}$).

^{13}C NMR (150.9 MHz, **d_7-DMF**, –**60 °C):** *δ* [ppm] = 36.1 (q, (*Z*)–$\underline{C}H_3$), 41.7 (q, (*E*)–$\underline{C}H_3$), 160.1 (d,–$\underline{C}$H).

^{15}N NMR (60.8 MHz, **d_7-DMF**, –**60 °C**, $^{15}N_6$-**7):** *δ* [ppm] = –162.50 (dt, ^1J(N_{imine}N-1) = 14.30 Hz, ^2J(N_{imine}N-2/N-5) = 2.21 Hz, N_{imine}), –59.58 (tdt, ^1J(N-1N-2/N-5) = 17.99 Hz, ^1J(N-1N_{imine}) = 14.30 Hz, ^2J(N-1N-3/N-4) = 0.84 Hz, N-1), –32.61 (m, N-2/N-5), 1.89 (m, N-3/N-4).

The assignment of the methyl-groups was done via NOE-experiments.

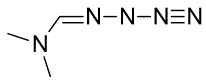

17

^{1}H NMR (600 MHz, **d_7-DMF**, –**60 °C):** *δ* [ppm] = 2.74 (s, 3H, –C$\underline{H}_3$), 2.92 (s, 3H, –C$\underline{H}_3$), 7.73 (d, 1H, –C$\underline{H}$).

^{13}C NMR (150.9 MHz, **d_7-DMF**, –**60 °C):** *δ* [ppm] = 34.4 (q, –$\underline{C}H_3$), 40.3 (q, –$\underline{C}H_3$), 154.4 (d,–$\underline{C}$H).

^{15}N NMR (60.8 MHz, **d_7-DMF**, –**60 °C**, $^{15}N_4$-**17):** *δ* [ppm] = –245.32 (dd, $^1J(N_\alpha N_\beta)$ = 14.6 Hz, $^1J(N_\alpha N_{imine})$ = 10.3 Hz, N_α), –147.69 (ddd, $^1J(N_{imine}N_\alpha)$ = 10.3 Hz, $^2J(N_{imine}N_\beta)$ = 3.8 Hz, $^3J(N_{imine}N_\gamma)$ = 1.7 Hz, N_{imine}), –116.37 (ddd, $^1J(N_\beta N_\alpha)$ = 14.6 Hz, $^1J(N_\beta N_\gamma)$ = 9.8 Hz, $^2J(N_\beta N_{imine})$ = 3.8 Hz, N_β), –112.68 (dd, $^1J(N_\gamma N_\beta)$ = 9.8 Hz, $^1J(N_\gamma N_{imine})$ = 1.7 Hz, N_γ).

Determination of the half-life

Previous experiments, as described at the top of this page, roughly showed that a first gas evolution in the course of this reaction occurred visibly at ca. –37 °C and a second one started at ca. +15 °C. ^{15}N NMR spectra indicated after keeping the NMR tube mentioned above at 0°C for 40 min, that only the signals of compound **17** disappeared. To determine the half-life of both species **7** and **17**, the following procedure was used:

In a Schlenk tube, pure **6** (250 mg, 2 mmol) was dissolved in anhydrous DMF (8 mL) and cooled under argon to –60 °C. A suspension of sodium azide (273 mg, 4.2 mmol, 2.1 eq.) in anhydrous DMF (2 mL) was added in one portion. The Schlenk tube was immediately closed and connected to a pneumatic apparatus. The cooling bath was exchanged with a cooling bath tempered to –30 °C. The collected gas volume in this step was 85 mL and half of it was collected in 16 min. Tempering the cooling bath to +21 °C leads to a second gas evolution with an additional gas volume of 58 mL – half-life: 11 min. The total gas volume was 143 mL (expected: 144 mL; yield 99%).

6. Spectra

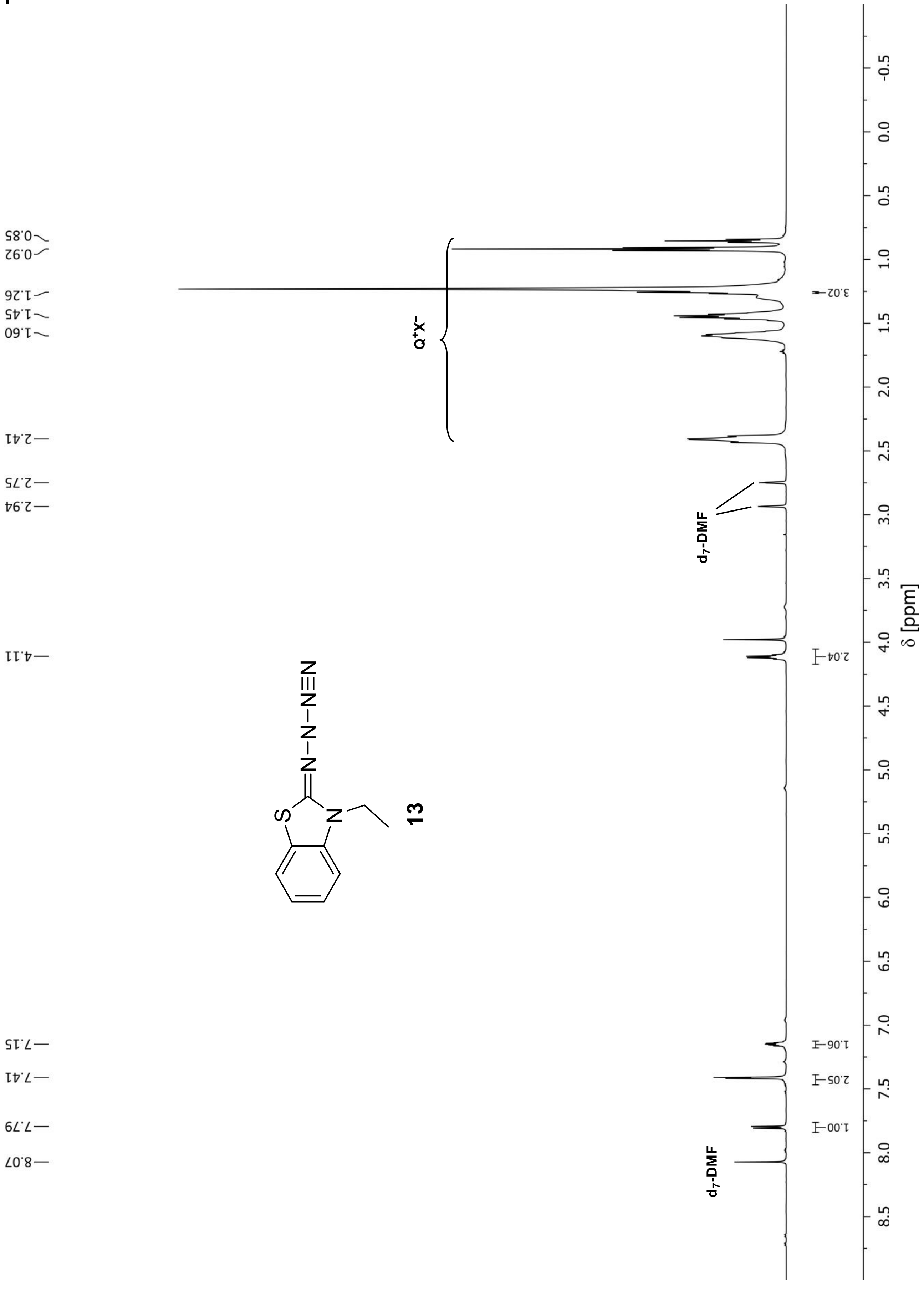

^{1}H NMR spectrum of $^{15}N_3$-**13** in d_7-DMF at −60 °C (4.1. method a)

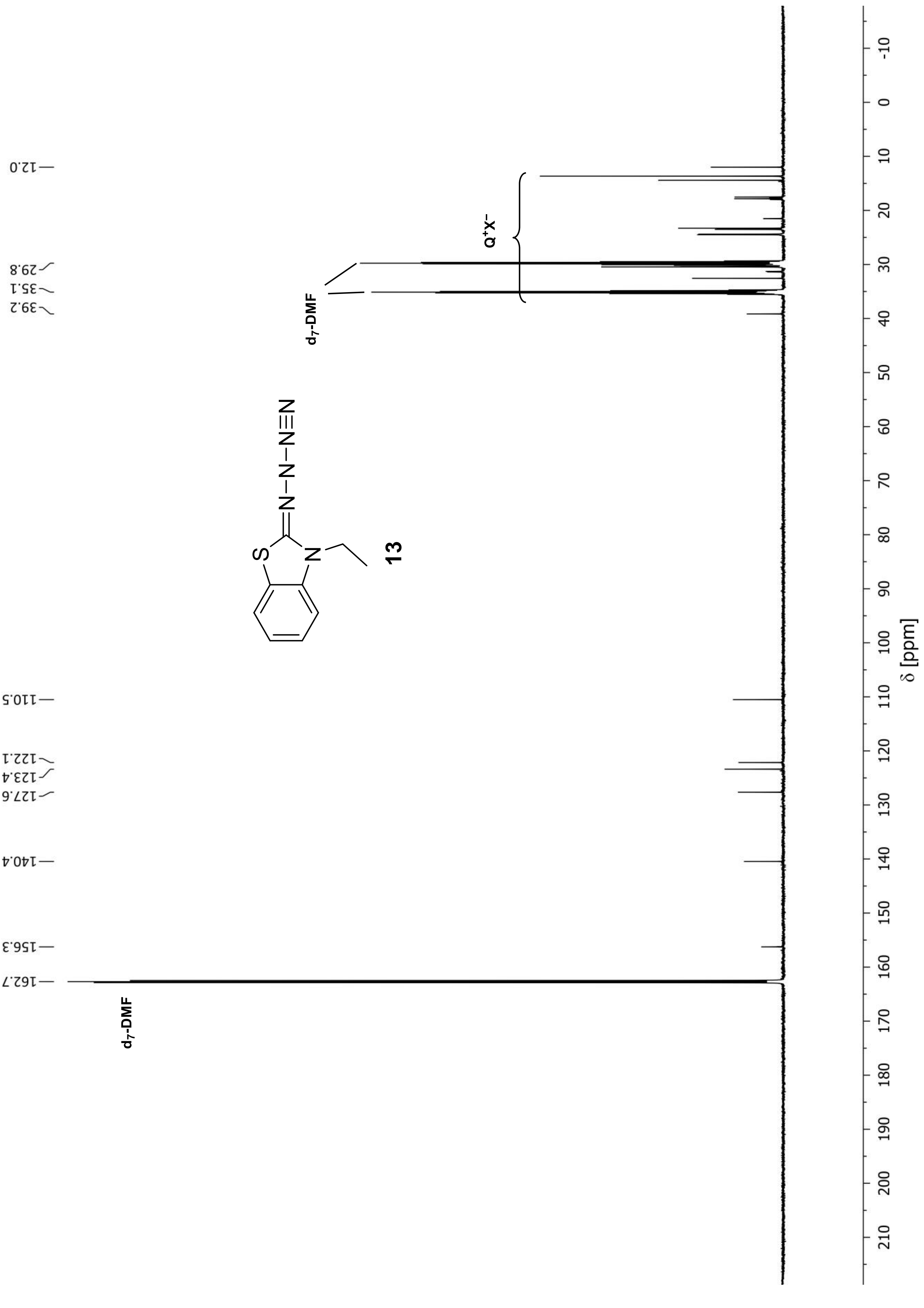

^{13}C NMR spectrum of $^{15}N_3$-**13** in d_7-DMF at −60 °C (4.1. method a)

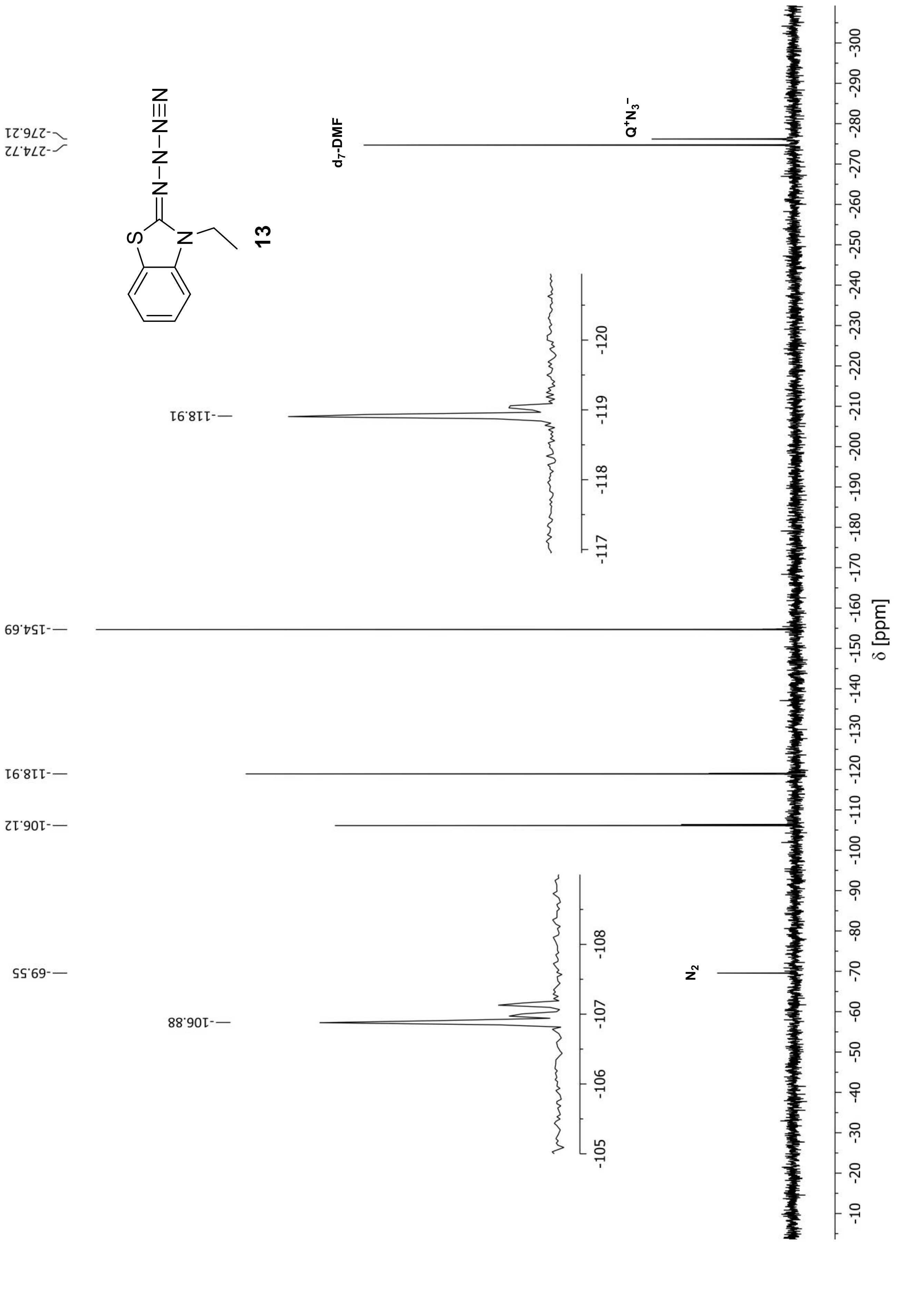

^{15}N NMR spectrum of $^{15}N_3$-**13** in d$_7$-DMF at −60 °C (4.1. method a)

^{15}N NMR spectrum of $^{15}N_4$-**13** in d_7-DMF at –60 °C (4.1. method b)

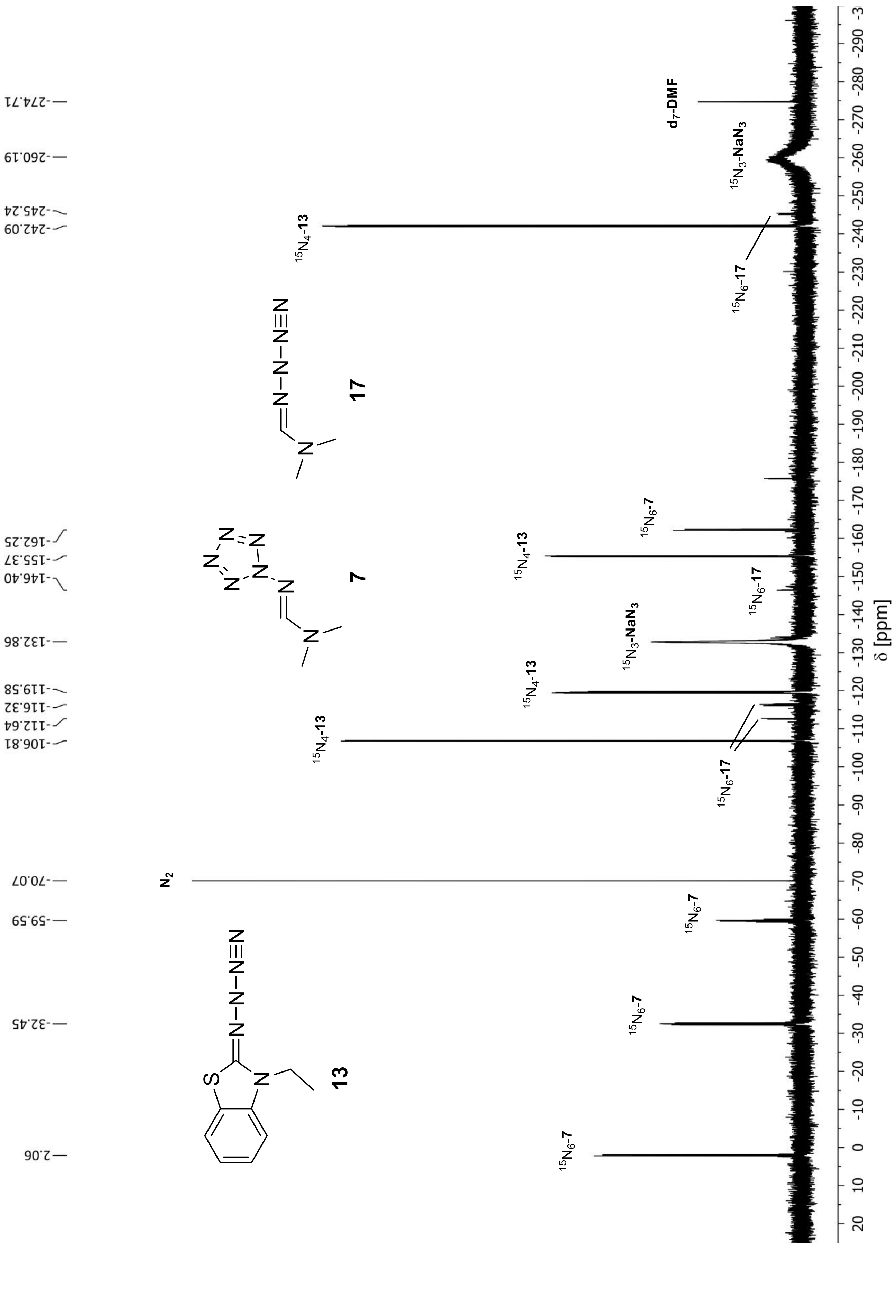

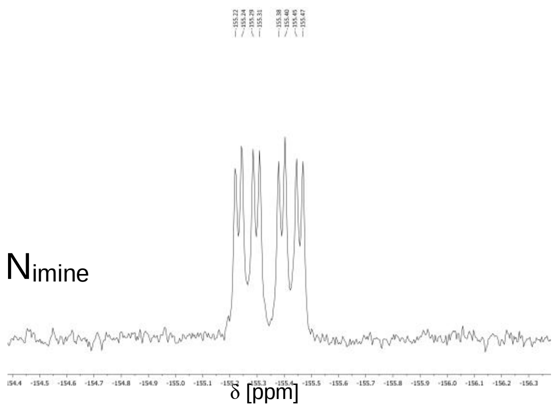

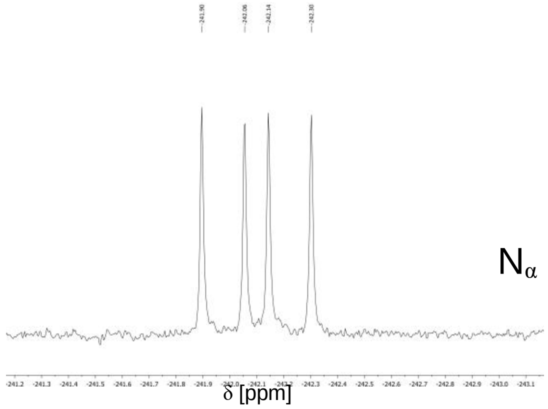

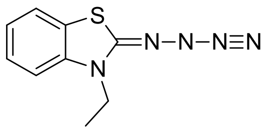

$^{15}N_4$-**13**

(magnified ^{15}N NMR spectrum, d_7-DMF, –60 °C, 4.1. method b)

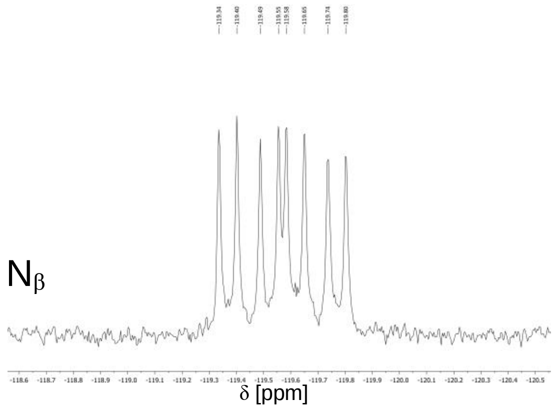

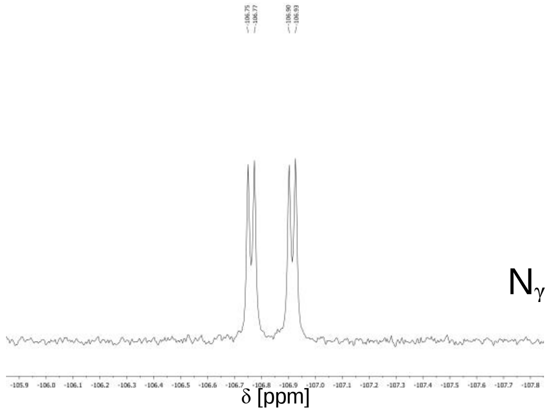

^{1}H NMR spectrum of $^{15}N_4$-**14** in d_7-DMF at rt (4.3. method c)

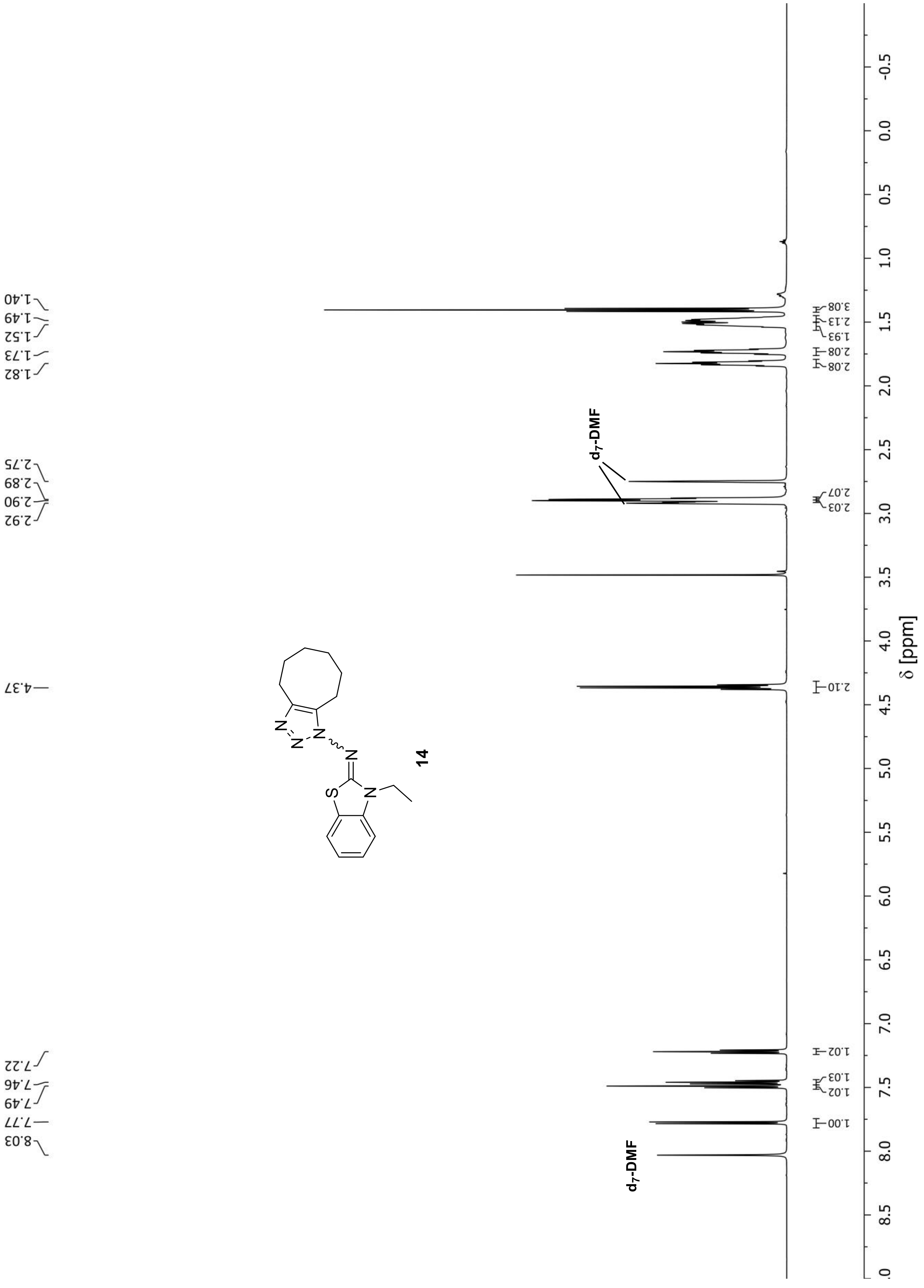

^{13}C NMR spectrum of $^{15}N_4$-**14** in d_7-DMF at rt (4.3. method c)

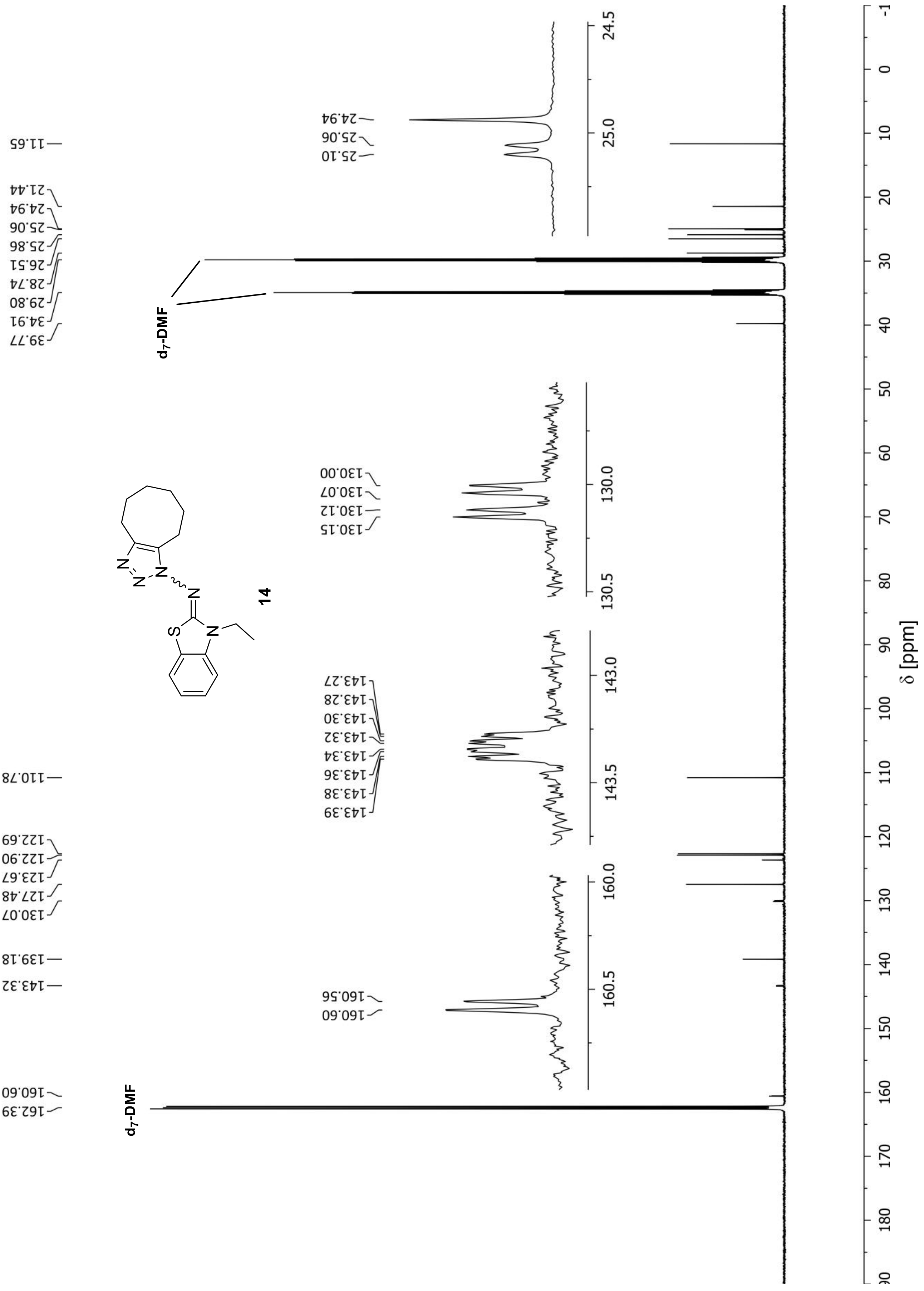

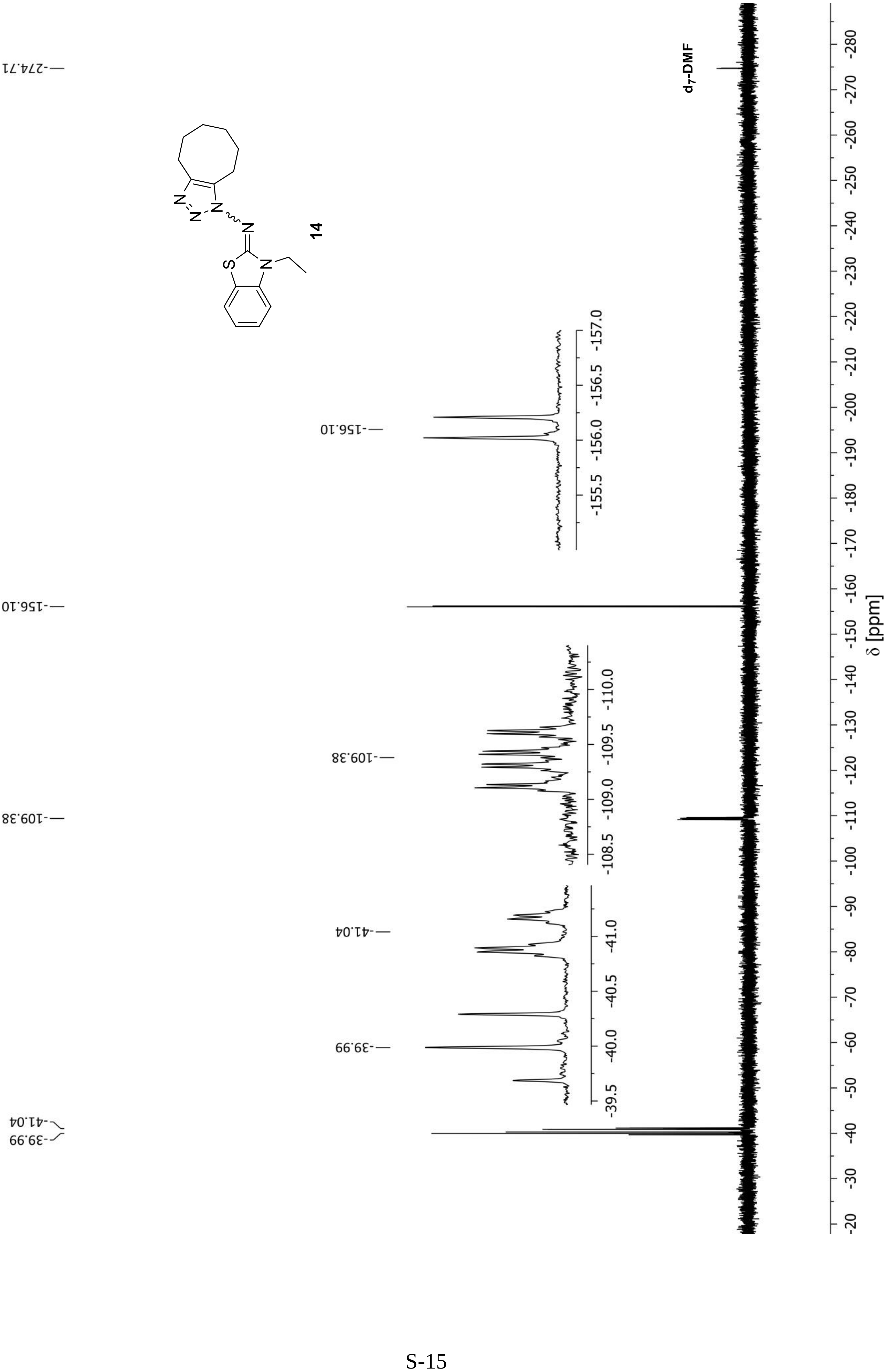

^{15}N NMR spectrum of $^{15}N_4$-**14** in d$_7$-DMF at rt (4.3. method c)

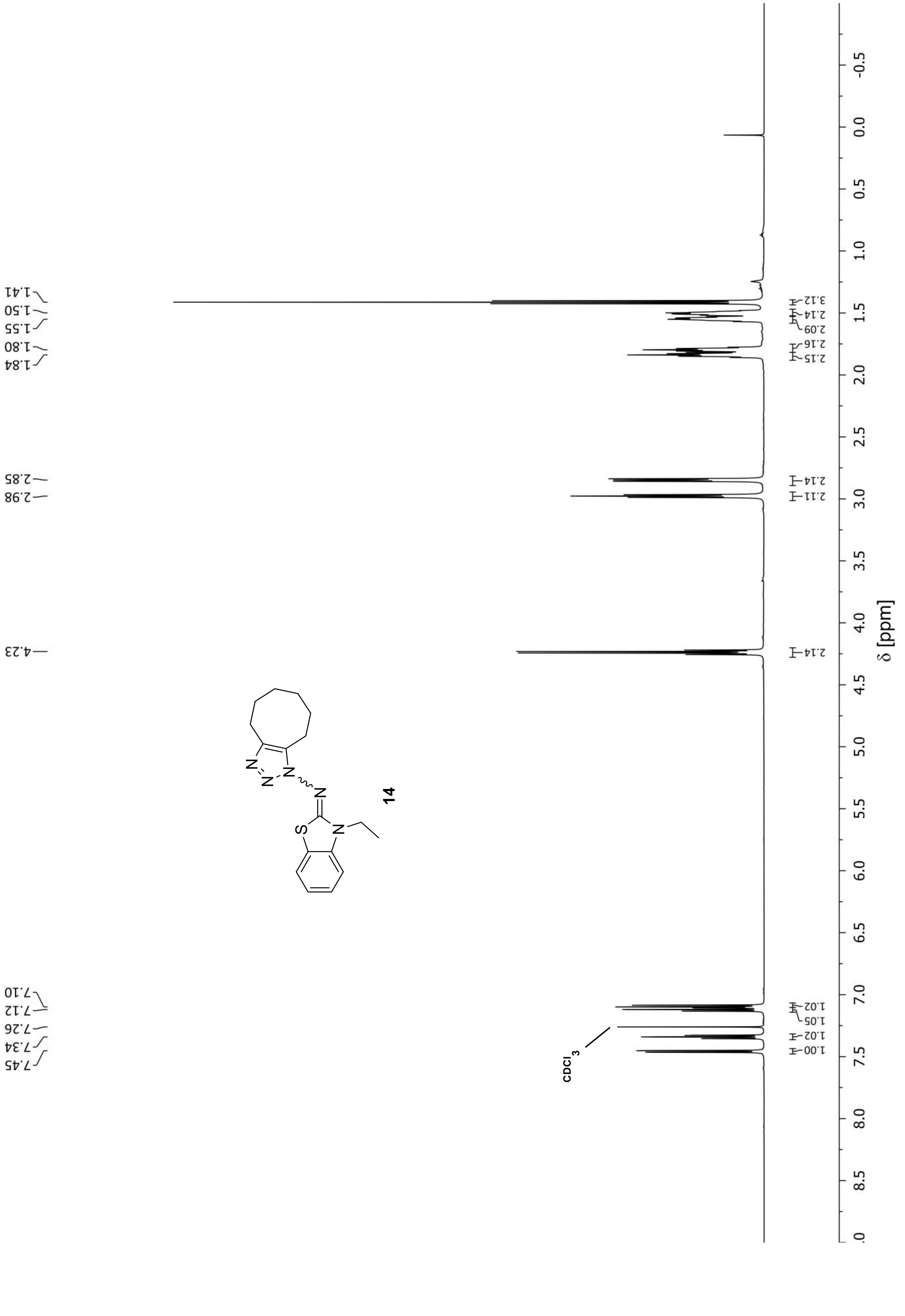

^{1}H NMR spectrum of **14** in $CDCl_3$ at rt (4.3. method b)

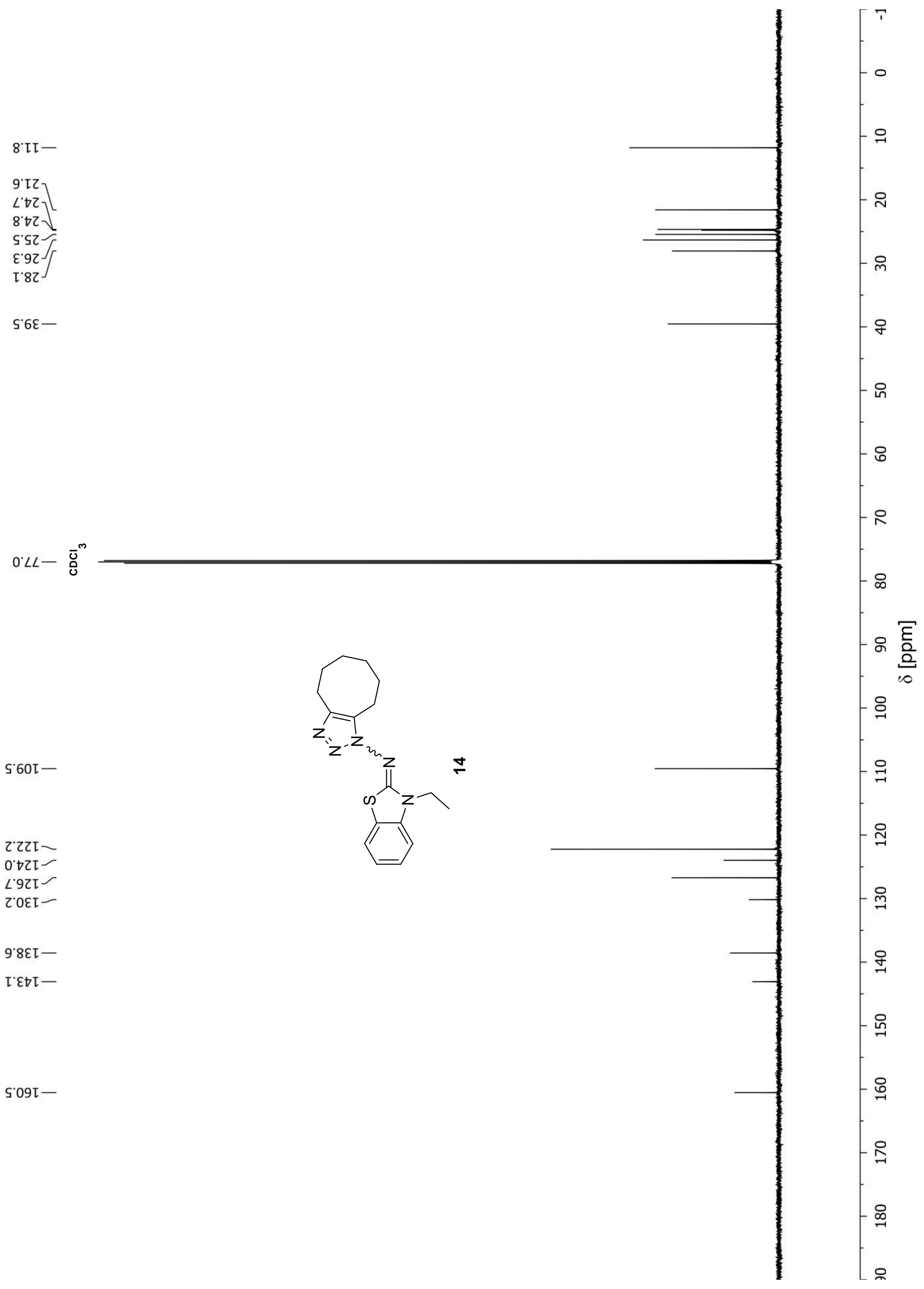

^{13}C NMR spectrum of **14** in $CDCl_3$ at rt (4.3. method b)

^{15}N NMR spectrum of **14** in $CDCl_3$ at rt (4.3. method b)

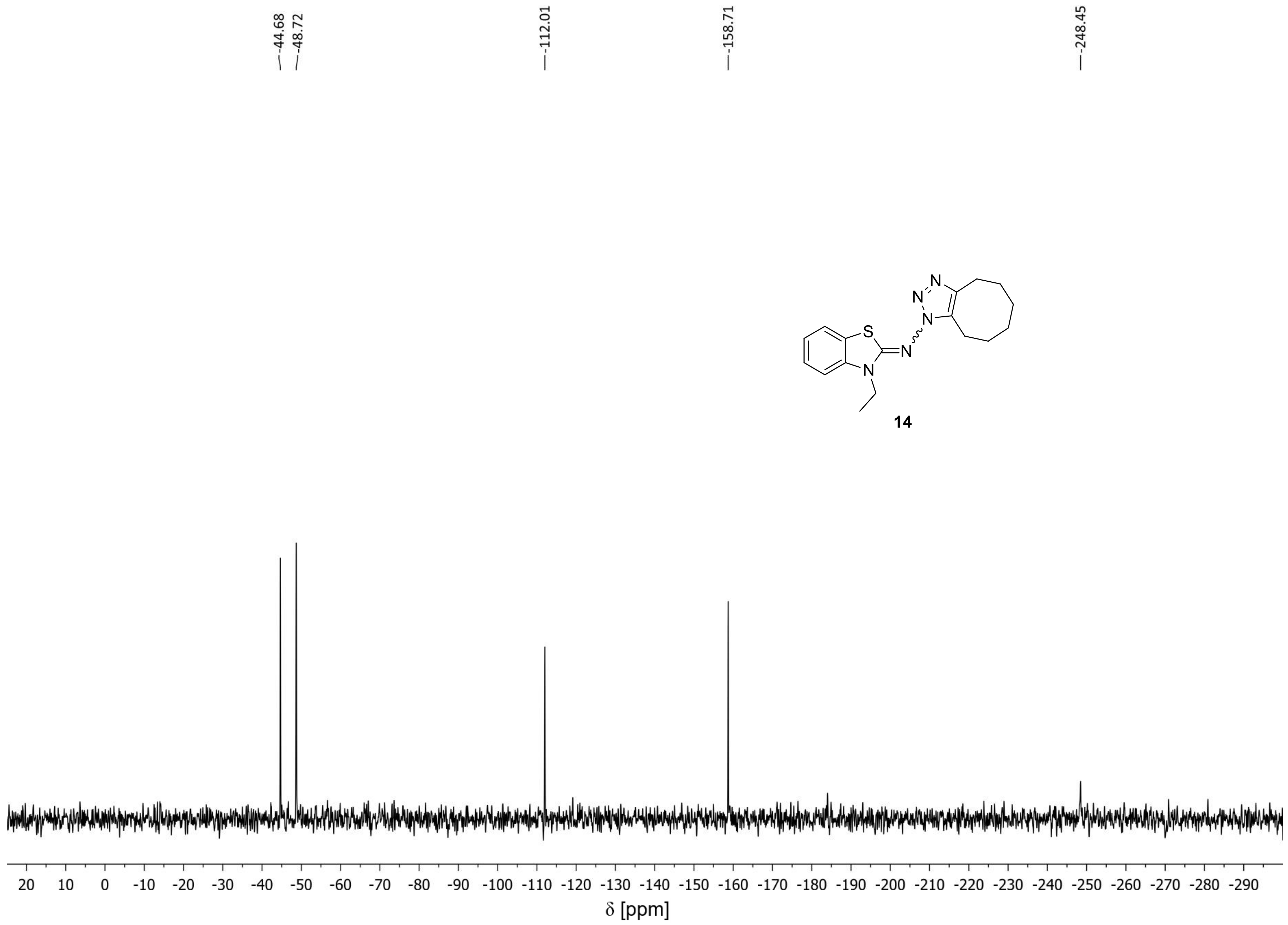

^{15}N NMR spectrum of $^{15}N_3$-**14** in $CDCl_3$ at rt (4.3. method a)

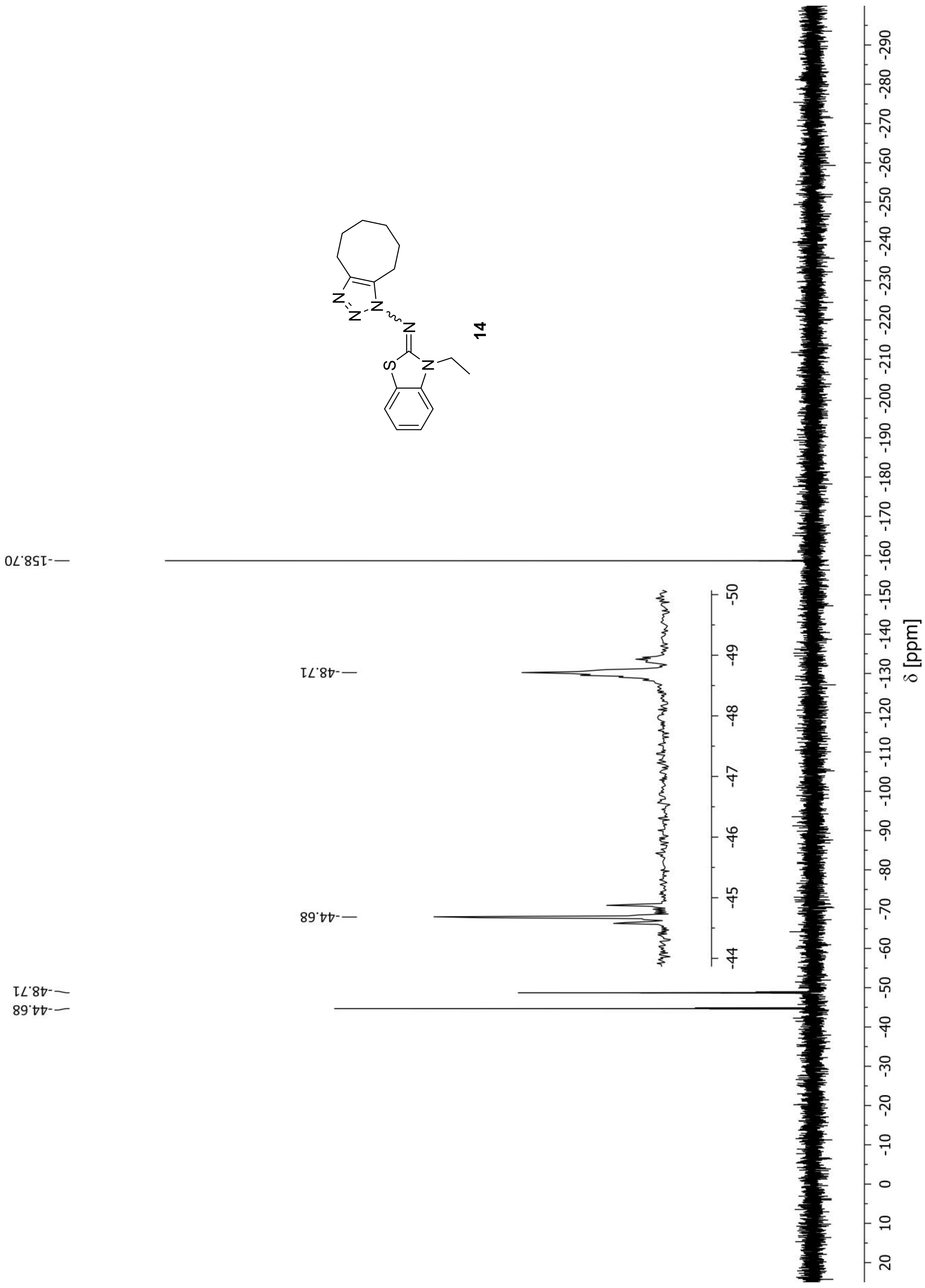

^{15}N NMR spectrum of $^{15}N_4$-**14** in $CDCl_3$ at rt (4.3. method c)

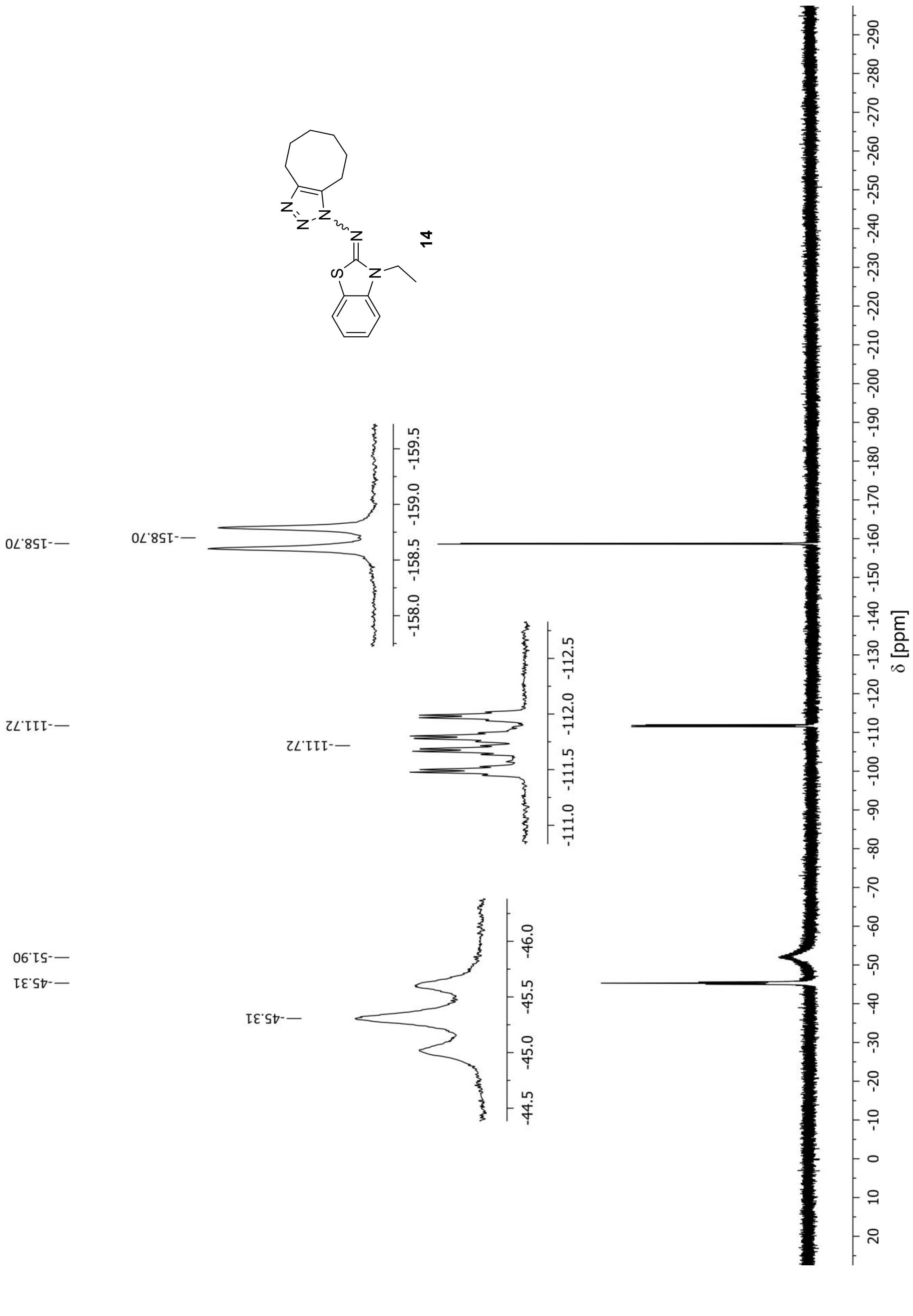

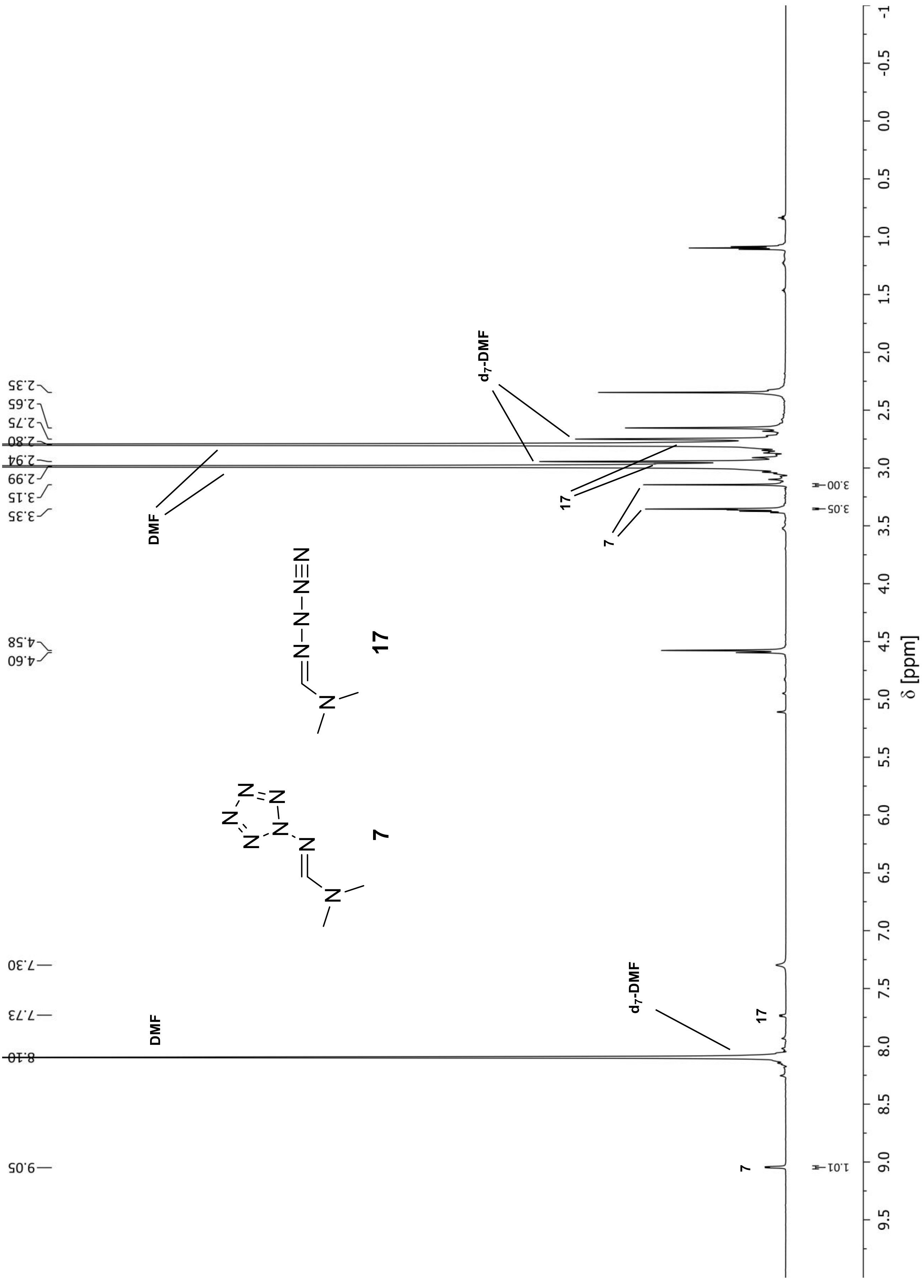

^{1}H NMR spectrum of $^{15}N_6$-**7** and $^{15}N_4$-**17** in d_7-DMF at –60 °C (5.)

^{13}C NMR spectrum of $^{15}N_6$-**7** and $^{15}N_4$-**17** in d_7-DMF at –60 °C (5.)

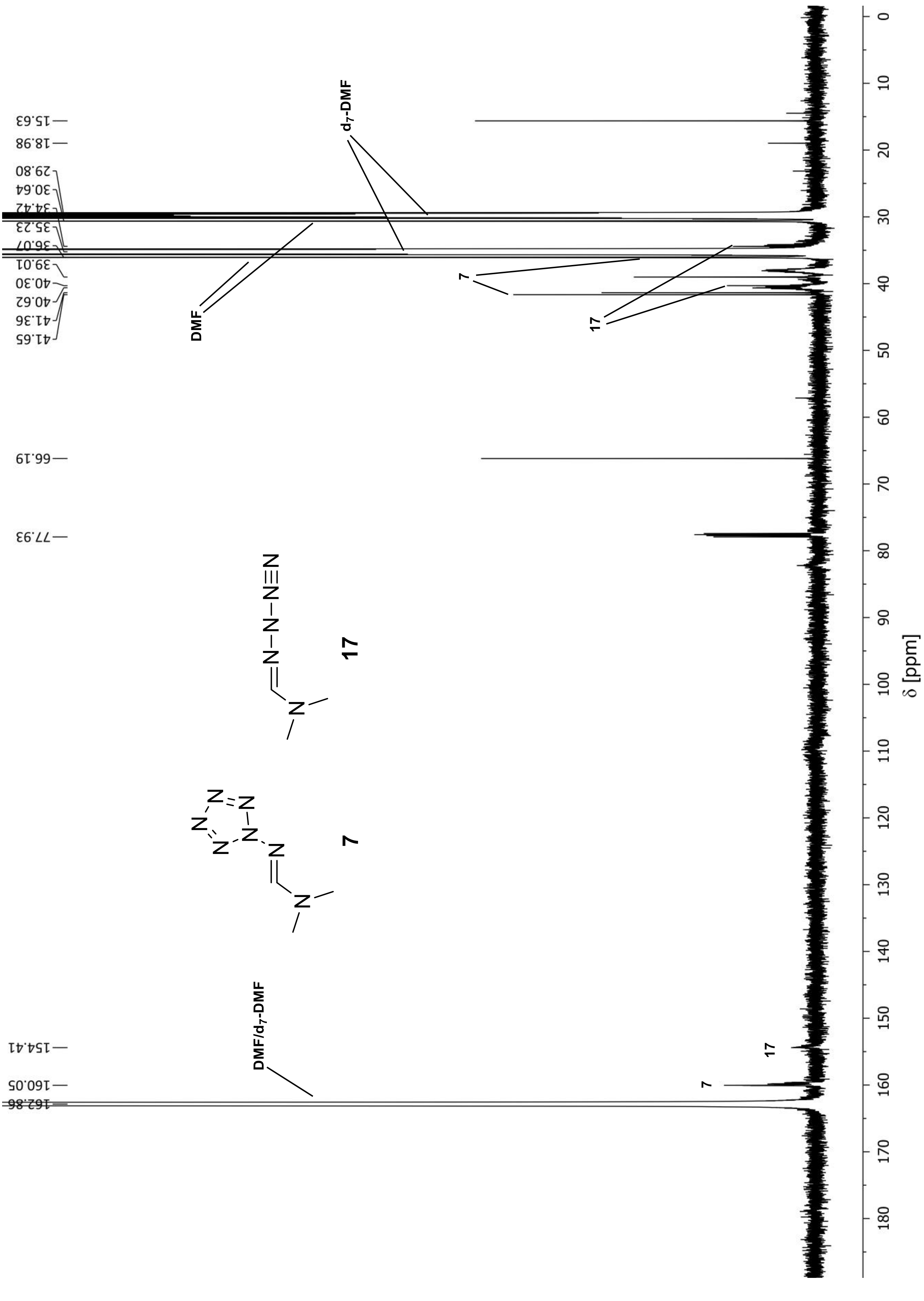

^{15}N NMR spectrum of $^{15}N_6$-**7** and $^{15}N_4$-**17** in d_7-DMF at −60 °C (5.)

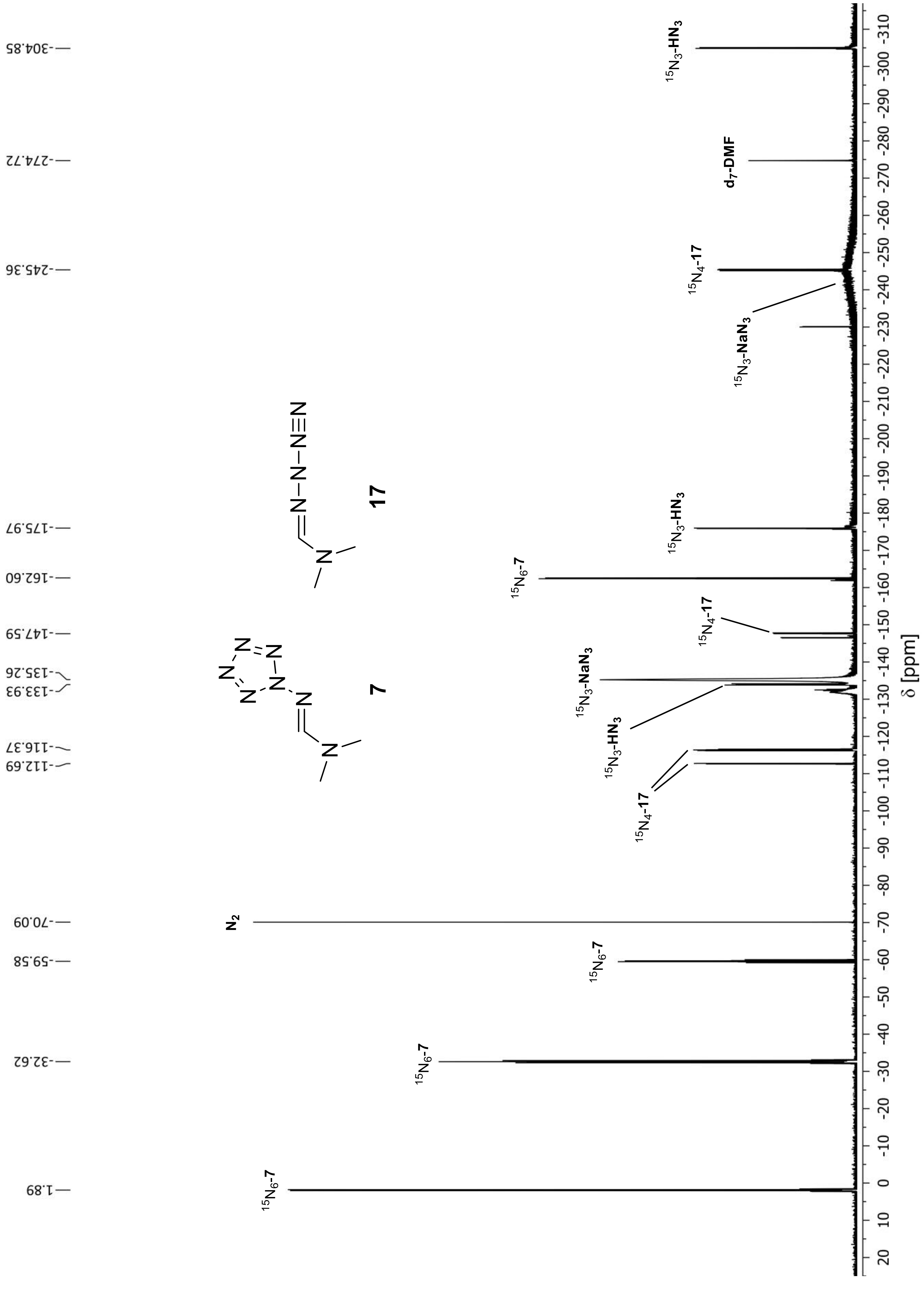

^{15}N NMR spectrum of $^{15}N_4$-**7** and $^{15}N_3$-**17** in DMF (d_2-DCM for lock in capillary) at –60 °C (5.)

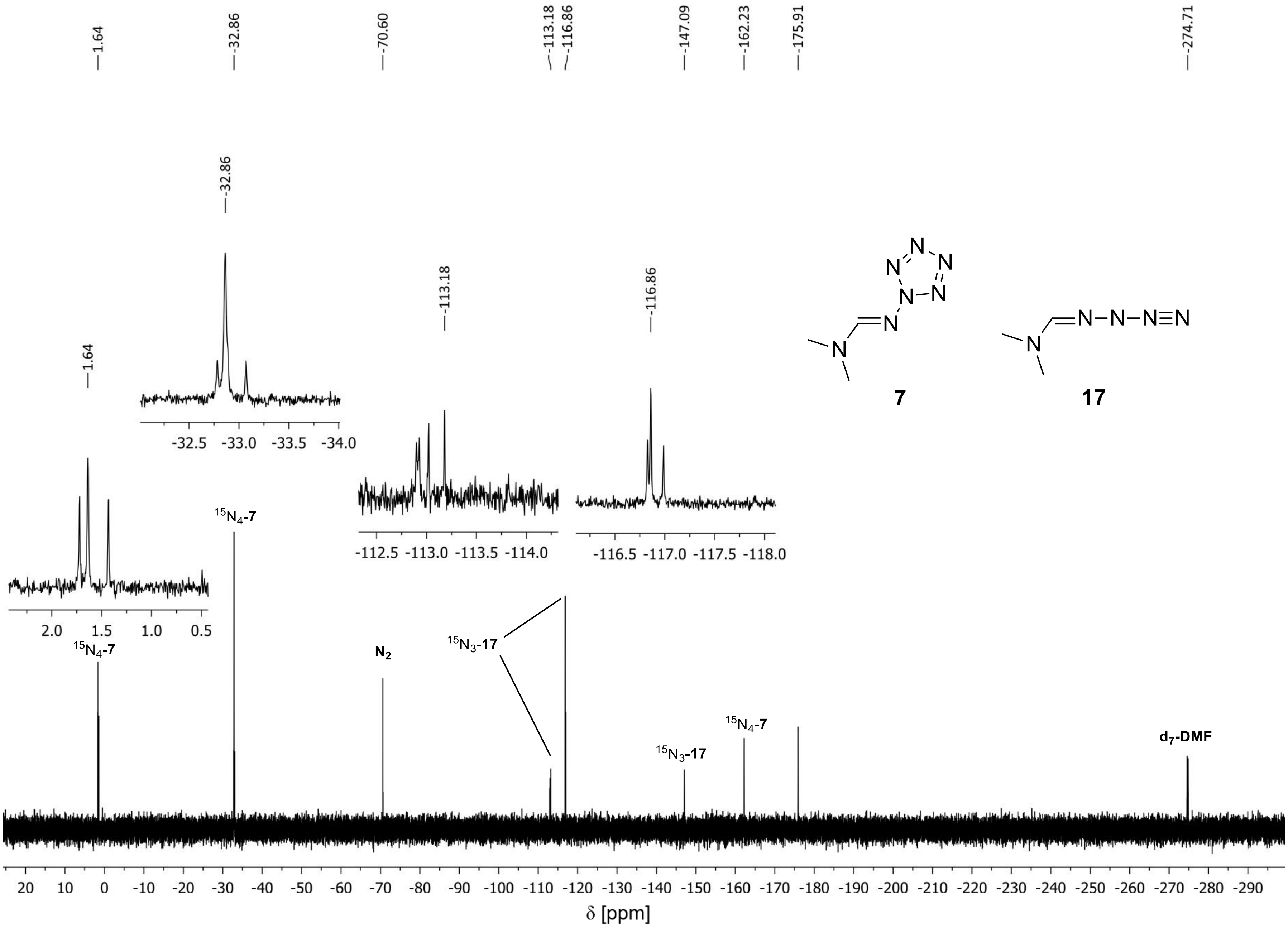

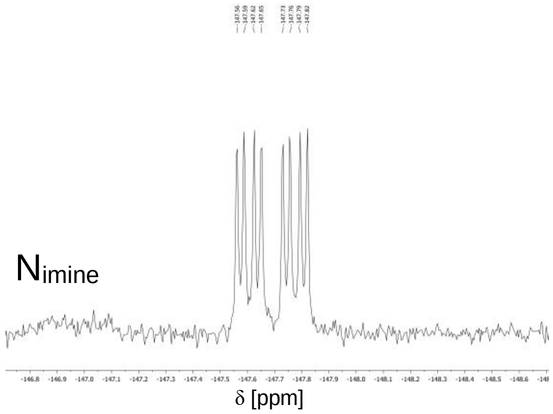

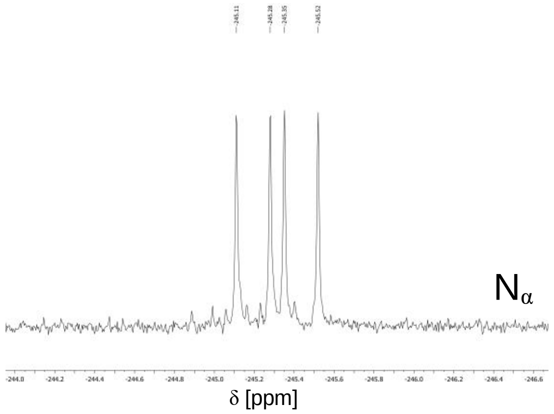

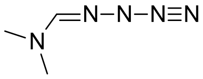

$^{15}N_4$-**17**

(magnified ^{15}N NMR spectrum, d_7-DMF, –60 °C, 5.)

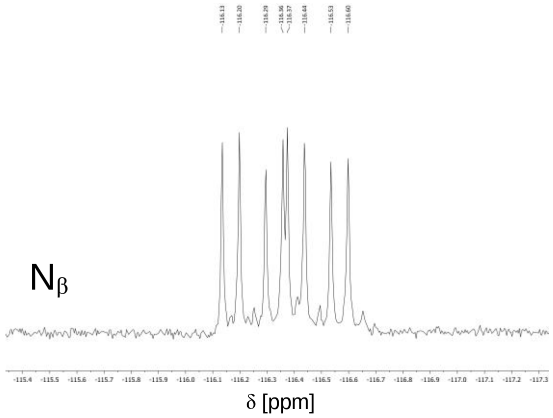

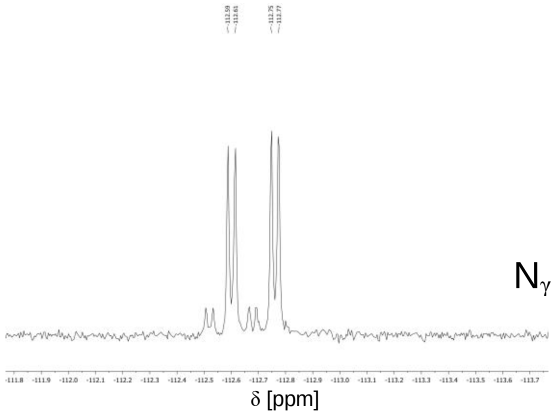

7. References

[S-1] K. Banert, *Chem. Ber.* **1985**, *118*, 1564–1574.

8. Author Contributions

T.P. performed the experimental work and prepared the Supporting Information. K.B. wrote the manuscript.
